# A Product of Heme Catabolism Modulates Bacterial Function and Survival

**DOI:** 10.1371/journal.ppat.1003507

**Published:** 2013-07-25

**Authors:** Christopher L. Nobles, Sabrina I. Green, Anthony W. Maresso

**Affiliations:** Baylor College of Medicine, Department of Molecular Virology and Microbiology, Houston, Texas, United States of America; Vanderbilt University, United States of America

## Abstract

Bilirubin is the terminal metabolite in heme catabolism in mammals. After deposition into bile, bilirubin is released in large quantities into the mammalian gastrointestinal (GI) tract. We hypothesized that intestinal bilirubin may modulate the function of enteric bacteria. To test this hypothesis, we investigated the effect of bilirubin on two enteric pathogens; enterohemorrhagic *E. coli* (EHEC), a Gram-negative that causes life-threatening intestinal infections, and *E. faecalis*, a Gram-positive human commensal bacterium known to be an opportunistic pathogen with broad-spectrum antibiotic resistance. We demonstrate that bilirubin can protect EHEC from exogenous and host-generated reactive oxygen species (ROS) through the absorption of free radicals. In contrast, *E. faecalis* was highly susceptible to bilirubin, which causes significant membrane disruption and uncoupling of respiratory metabolism in this bacterium. Interestingly, similar results were observed for other Gram-positive bacteria, including *B. cereus* and *S. aureus*. A model is proposed whereby bilirubin places distinct selective pressure on enteric bacteria, with Gram-negative bacteria being protected from ROS (positive outcome) and Gram-positive bacteria being susceptible to membrane disruption (negative outcome). This work suggests bilirubin has differential but biologically relevant effects on bacteria and justifies additional efforts to determine the role of this neglected waste catabolite in disease processes, including animal models.

## Author Summary

Bilirubin is the terminal breakdown product of heme, which is deposited at high concentrations in the human intestine, where it can come into contact with host cells, the gastrointestinal (GI) microflora, and invading pathogens. Here, we report that bilirubin can act as a protectant for the Gram-negative bacterial pathogen *E. coli* O157:H7, which causes severe hemorrhagic diarrhea and life-threatening kidney damage. Paradoxically, bilirubin is highly toxic towards another enteric opportunistic pathogen, the Gram-positive bacterium *E. faecalis*. Whereas the protection of *E. coli* stems from the neutralization of host reactive oxygen species, bilirubin's toxicity toward *E. faecalis* is rooted in its lipophilic properties, which drives the rapid association of bilirubin with bacteria, leading to disrupted cell membranes and concomitant death. These results suggest small molecule metabolites can modulate bacterial communities in the intestine, a finding that may have important implications for diseases caused by enteric bacteria and disrupted flora.

## Introduction

Heme is a critical co-factor in aerobic respiration and energy production, yet in excess is also highly toxic [1]. The turnover and degradation of heme is a protective pathway, terminating in the production of bilirubin [1], [2], [3]. Heme is broken down predominately in the spleen by heme oxygenase 1 and 2 into molecular iron, carbon monoxide, and a green pigment called biliverdin, which is further reduced by biliverdin reductase to yield bilirubin [4]. Lipophilic bilirubin is transported by serum via albumin to the liver and removed from circulation. Biotransformation of bilirubin from a lipophilic molecule to water-soluble or conjugated forms (bilirubin mono- and di-glucuronide) is facilitated by the 1A1 isoform of uridine 5′-diphosphate-glucuronosyltransferase (UGT1A1) [4]. After conjugation with glucuronic acid, bilirubin is actively secreted across the canalicular membrane of hepatocytes by MRP2 and into bile [5]. Host and bacterially secreted β-glucuronidases, as well as non-enzymatic hydrolysis, lead to the deconjugation of bilirubin glucuronide after bile is released into the intestine, resulting in unconjugated bilirubin that can be found in the intestinal lumen of humans at submillimolar concentrations [6], [7]. Approximately 300 mg of bilirubin are produced daily by healthy adults, the vast majority of which is excreted in feces [8], [9], [10].

Bilirubin affects mammalian systems in diverse ways. In a series of elegant experiments, Stocker *et* al. demonstrated bilirubin can scavenge peroxyl radicals and other reactive oxygen species (ROS) [2], [10]. *In vivo*, bilirubin decreased injury from hyperoxia in neonatal Gunn rats [11]. In population studies, high baseline serum bilirubin concentrations correlated significantly with reduced rates of cancer-related mortality, suggesting bilirubin may alleviate the oxidative stress contributing to carcinogenesis [12], [13]. In excessive quantities, however, bilirubin can enter the central nervous system, leading to encephalitis. This associated toxicity has been previously described in both neural and non-neural cell lines, leading to decreased neuronal cell viability and hemolysis of circulating erythrocytes [14]. However, none of these effects have any direct connection to the site in the body where bilirubin is heavily deposited, the GI tract.

ROS in the GI tract is generated by three main sources, including infiltrating phagocytes, the intestinal epithelium, and the resident commensal bacteria. An essential factor to kill engulfed bacteria, infiltrating phagocytes utilize NADPH-dependent systems of generating superoxide called the NADPH oxidase (Nox) family of proteins, which oxidize NADPH to NADP+ in order to transfer electrons to molecular oxygen. The oxidation of NADPH to generate ROS, termed the “respiratory burst” mechanism, is also utilized by dual oxidase (Duox) family of proteins, which are expressed on the apical plasma membrane of GI epithelia cells and generate hydrogen peroxide into the luminal environment. The generation of hydrogen peroxide can further assist in the generation of antimicrobial molecules by interacting with lactoperoxidase to form hypothiocyanite ions [15]. In addition, intestinal commensal bacteria can produce ROS. A system for production of extracellular ROS has been described for the Gram-positive bacterium *Enterococcus faecalis*, which generates extracellular superoxide through the autoxidation of demethylmenaquinone, and is implicated in the damaging of colonic epithelial DNA [16]. Superoxide production by *E. faecalis* has been postulated to provide a competitive advantage for growth in the intestinal ecosystem; however, this has not been proven.

Postulating that this somewhat neglected heme catabolite bilirubin can form functional interactions with intestinal bacteria, we characterized the effect of this pigment on the GI bacteria. We chose to utilize a Gram-negative intestinal pathogen (enterohemorrhagic *Escherichia coli*) which can cause severe diarrhea and life-threatening kidney damage, and a Gram-positive opportunistic pathogen (*E. faecalis*) known for their increasing broad-spectrum antibiotic resistance [17], to study the effects of bilirubin on two enteric pathogens. Our data reveal, for the first time, that bilirubin can dramatically alter bacterial biology, enhancing on one hand pathogen survival while on the other causing a disruption of cell integrity. These results have wide-spread implications for those who study host-pathogen and host-commensal homeostasis in the human intestine.

## Results

### Whole bile contains antioxidant activity

**Figure 1:**
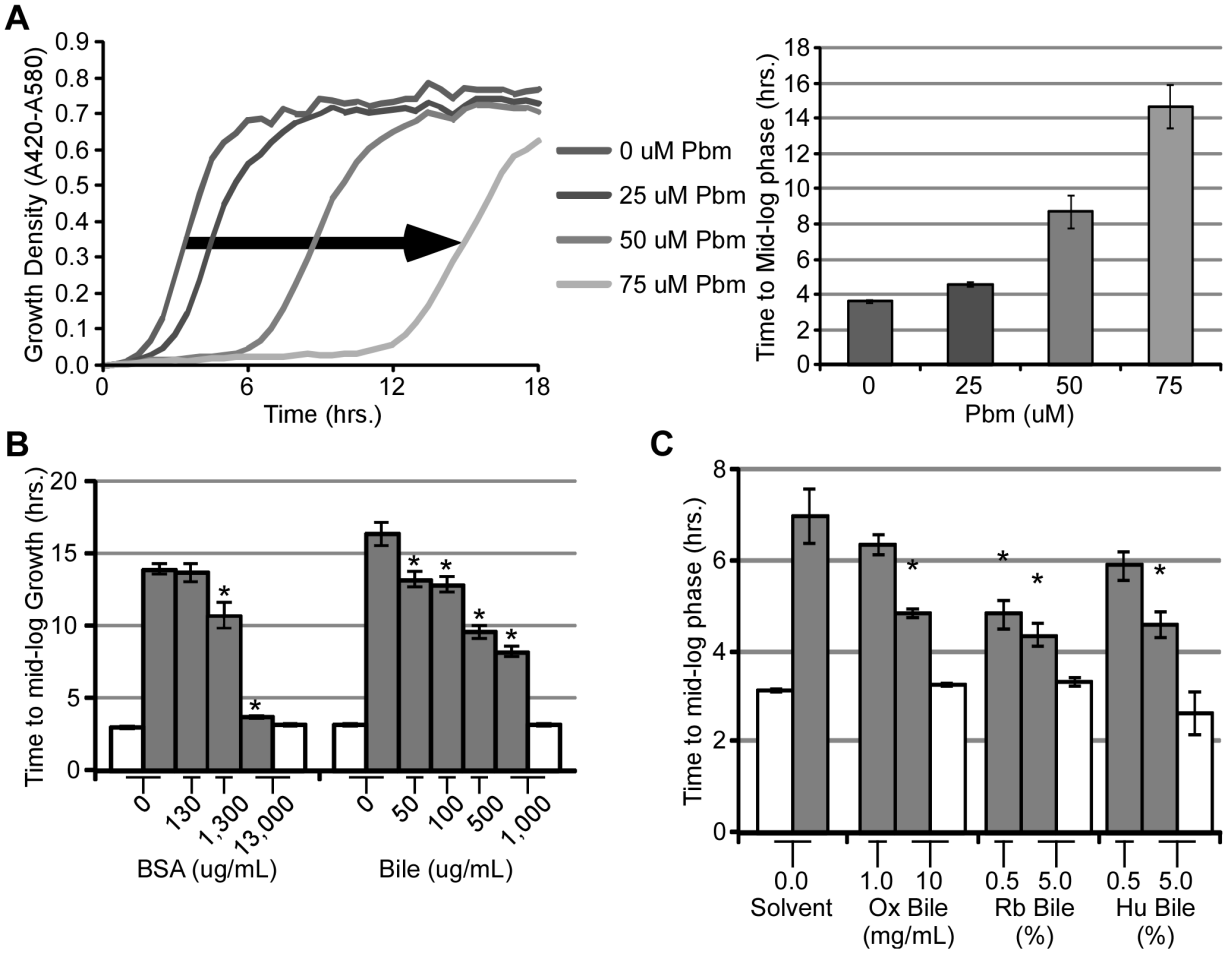
The effect of bile on EHEC growth in the presence of ROS. (A, Left) Wideband absorbance (420–580 nm) of EHEC (EDL933) cultures supplemented with plumbagin (0, 25, 50, and 75 µM) was monitored while cultures were grown at 37°C with shaking. (A, Right) The time to mid-log phase of each culture was calculated from the growth curves. (B) EHEC (EDL933) cultures supplemented with (grey bars) or without (white bars) plumbagin (50 µM) and/or BSA (2, 20, and 200 uM BSA) and/or ox bile (50, 100, 500, 1000 ug/mL ox bile). (C) EHEC (86-24) cultures were supplemented with plumbagin (50 µM) (grey bars) or without plumbagin (white bars) and either ox, rabbit (Rb), or human (Hu) bile (1 and 10 mg/mL ox bile; 0.5 and 5.0% rabbit and human bile). Error bars represent ± one standard deviation, n = 3, and (*) denotes a significant (P≤0.05) difference between treated samples and solvent-treated samples.

We formulated the simple hypothesis that bile, because of its high concentration of bilirubin, may act as an antioxidant and neutralize free radicals. To test this hypothesis, cultures of *E. coli* serotype O157:H7 (EHEC), an outbreak strain that can cause life-threatening intestinal infections, was supplemented with the quinone plumbagin. Membrane-associated quinones, like plumbagin, generate ROS by shuttling electrons from the electron transport system to molecular oxygen, thereby generating oxygen radicals such as superoxide, which can kill cells [18]. The addition of plumbagin to EHEC cultures increased the time to mid-log phase in a dose-dependent manner, suggesting plumbagin, presumably through the generation of ROS, severely inhibited bacterial growth (Fig. 1A). Addition of bovine serum albumin (BSA) to EHEC cultures containing plumbagin decreased the time to mid-log phase in a dose-dependent manner (Fig. 1B). No effect on the culture growth was observed with the addition of BSA in the absence of plumbagin. This is consistent with the notion that BSA, a known antioxidant, was mitigating the negative effects of plumbagin by protecting from ROS. When EHEC was grown in the presence of plumbagin and ox bile, the time to mid-log phase decreased in a dose-dependent manner when compared to the absence of bile, an effect dependent on the presence of the radical generator (Fig. 1B). This effect was also observed for bile from other species, including human (Fig. 1C). Interestingly, rabbit bile seemed to be the most effective, which is consistent with its nearly two-fold greater concentration (approximately 600 uM) of bilirubin when compared to bile from ox (approximately 250 uM bilirubin in 100 mg/mL whole bile) or human (approximately 400 uM bilirubin, quantification conducted according to [19]). Collectively, this data suggests plumbagin-mediated toxicity can be alleviated by mammalian bile, possibly by the heme catabolite bilirubin.

### Bilirubin protects EHEC from ROS

**Figure 2:**
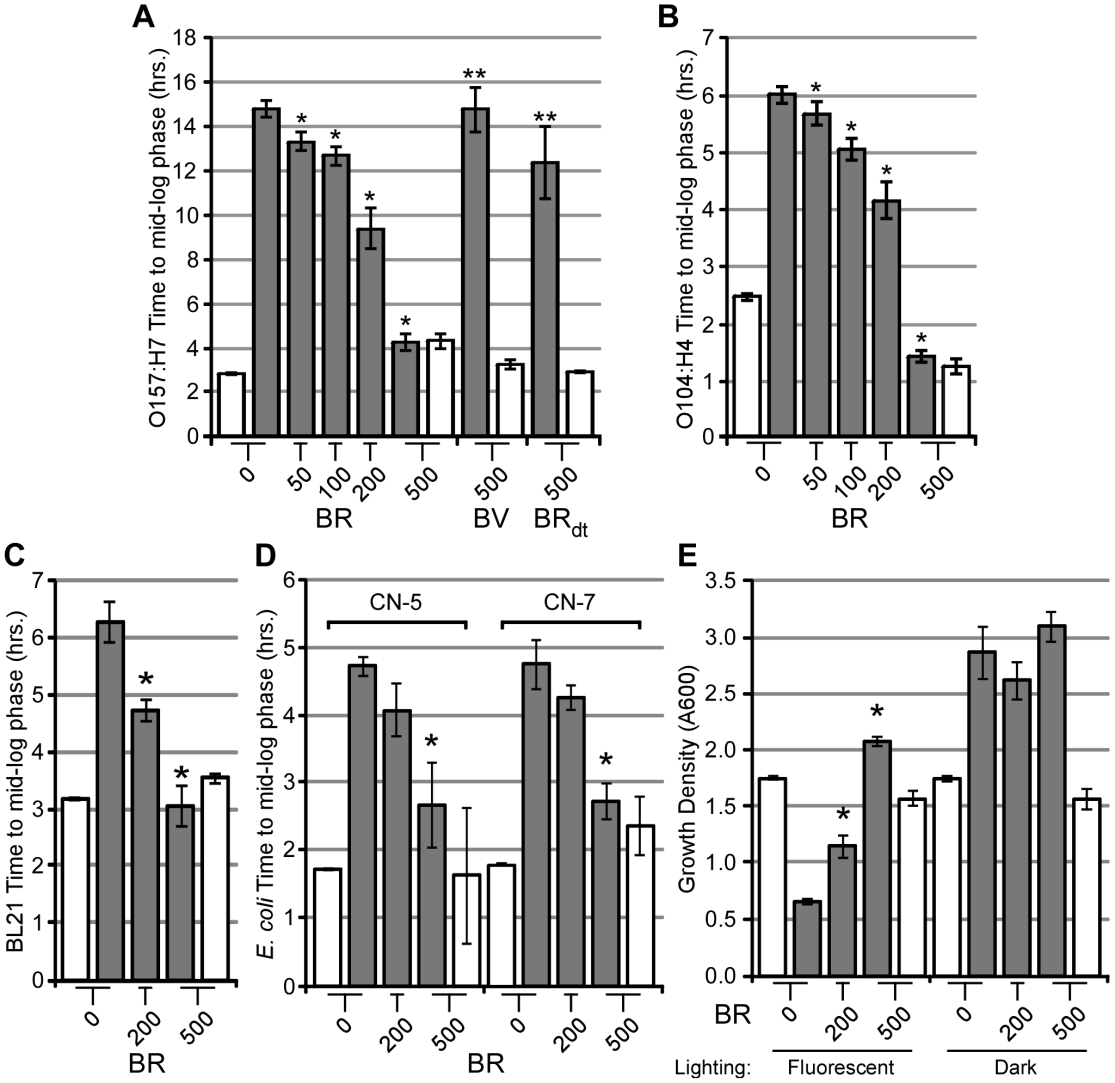
Bilirubin diminishes ROS-induced growth inhibition. EHEC O157:H7 (EDL933) (A), EAEC O104:H4 (B), or *E. coli* BL21 (C) cultures were supplemented with plumbagin (50 µM, grey bars) or without plumbagin (white bars) in the presence or absence of bilirubin (BR), biliverdin (BV), or bilirubin ditaurate (BR_dt_). (D) Commensal *E. coli* strains CN-5 and CN-7 were supplemented with plumbagin (75 µM, grey bars) or without plumbagin (white bars) in the presence or absence of bilirubin (BR). (E) EHEC strain 86-24 was supplemented with Rose Bengal (750 µM, grey bars) or solvent (white bars) with or without bilirubin (200 or 500 µM) and exposed to fluorescent lighting or contained in a dark box for 12 to 14 hours. Terminal culture growth density was quantified by measuring the absorbance at 600 nm. Error bars represent ± one standard deviation, n = 3, and (*) denotes a significant (P≤0.05) difference while (**) denotes a non-significant difference (P>0.05) between treated samples and solvent-treated samples.

We hypothesized that the protection of EHEC from plumbagin could be due to the bile pigments bilirubin or biliverdin. To test this hypothesis, we grew EHEC in the presence or absence of plumbagin with or without bilirubin. Interestingly, EHEC cultures exposed to ROS supplemented with bilirubin showed a dose-dependent reduction in the time to mid-log phase compared to those in the absence of bilirubin (Fig. 2A). Surprisingly, the addition of either biliverdin (the metabolic precursor to bilirubin) or bilirubin ditaurate (a more soluble synthetic form of bilirubin and substitute for bilirubin glucuronide) did not reduce the growth time of EHEC that was exposed to ROS, suggesting little to no antioxidant potential for these two bile pigments. Since biliverdin has a reduced capacity to neutralize free radicals (based on its structure, biliverdin cannot donate its central hydrogen, leading to reduction of ROS, as easily as bilirubin – see Fig. S1) and bilirubin ditaurate (which is soluble and contains the conjugated system similar to bilirubin, and thus retains antioxidant potential) also lacks the ROS scavenging activity (both do not protect EHEC from ROS), these data suggest unconjugated bilirubin protects by direct association with bacteria. In support of this finding, when we exposed EHEC to bilirubin and fractionated the cells, we found approximately 1/3 of the total bilirubin was recovered in the soluble lysate, whereas the remaining bilirubin was found in the insoluble pellet after clearance of the lysate (Fig. S2 and data not shown). This data suggests that bilirubin associates with EHEC, likely through hydrophobic interactions with membranes, as has been previously observed for the interaction of bilirubin with eukaryotic cells [14]. Interestingly, expression of human biliverdin reductase (BVR) in EHEC, which should generate intracellular bilirubin when cultures are fed the precursor biliverdin, failed to rescue the bacteria from ROS generated by plumbagin (Fig. S3). This may suggest that bilirubin's antioxidant activity may occur at the bacterial surface, possibly the inner and/or outer membrane.

To determine if the observed protection from ROS was strain dependent, the growth of the 2011 outbreak strain EAEC O104:H4, which sickened over 3,000 people in Europe (∼50 deaths), was examined for growth when exposed to plumbagin and bilirubin. Indeed, EAEC was protected from ROS when bilirubin was added to the cultures (Fig. 2B) [20]. Similarly, *E. coli* BL21, a laboratory strain that is not considered a pathogen, was protected from ROS when supplemented with bilirubin in a dose-dependent manner (Fig. 2C). Further, *E. coli* isolated from a healthy donor's stool sample were likewise tested and found to be protected from ROS when cultured with bilirubin (Fig. 2D). Each of the strains displayed varying susceptibilities towards the toxic effects of plumbagin, possibly from differential regulation of ROS neutralizing factors. Yet, in each experiment, the addition of bilirubin decreased the amount of toxicity generated by plumbagin, suggesting bilirubin could act as an antioxidant for each strain. Finally, to strengthen the notion that bilirubin was relieving oxidant stress generated by plumbagin, we repeated the above experiments with EHEC using a photosensitive ROS-generating agent called Rose Bengal, which generates superoxide from molecular oxygen under fluorescent lighting [21]. Growth of EHEC strain 86-24 was inhibited in a light-dependent manner with Rose Bengal present, yet growth was rescued when increasing amounts of bilirubin were supplemented into the cultures (Fig. 2E). Collectively, these results strengthen the notion that bilirubin protects diverse strains of *E. coli* from exogenous ROS, possibly at the level of the bacterial membrane, and justify efforts to examine the utility of this effect in a more biologically relevant context.

### Bilirubin protects EHEC from killing by macrophages

**Figure 3:**
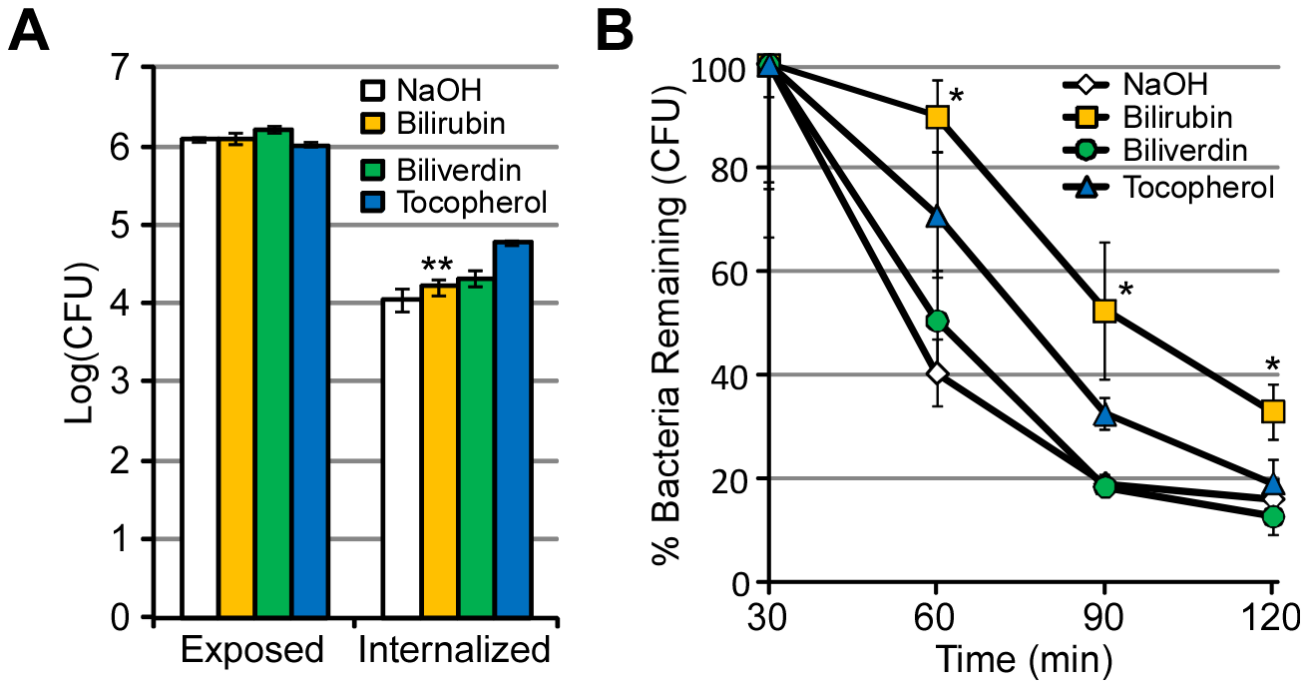
Bilirubin protects EHEC from killing by J774A.1 macrophages. EHEC (86-24) was cultured with solvent (white), bilirubin (orange, 250 µM), biliverdin (green, 250 µM) or α-tocopherol (blue, 250 µM), in minimal media for 6 hours before addition to macrophages (J774A.1) at an MOI of approximately 3. (A) The amount of EHEC exposed to the macrophages is compared to the amount internalized by macrophages after 30 minutes. (B) The percentage of internalized EHEC over 2 hours was monitored for bacteria cultured with solvent (white diamonds, NaOH), bilirubin (orange squares), biliverdin (green circles) and α-tocopherol (blue triangles). Error bars represent ± one standard deviation, n = 3. Data are representative of a single experiment repeated three times with similar results and the (*) denotes a significant (P≤0.05) difference while (**) denotes a non-significant difference (P>0.05) between treated samples and solvent-treated samples.

Macrophages kill bacteria after phagocytosis through the generation of superoxide by NADPH oxidase. This so-called “oxidative burst” is a major component of the host's native immunity against invading bacteria, including enteric pathogens in the intestine [15]. We hypothesized that bilirubin, due to its ability to protect EHEC from exogenous ROS (Fig. 2), may also mitigate ROS produced by macrophages during engulfment of EHEC. To test this hypothesis, we added EHEC strain 86-24 to J774A.1 murine macrophages at an MOI of approximately 3 and followed bacterial survival by plating the colony forming units (CFUs) of EHEC over time on selective agar post osmotic lysis of macrophages. Macrophages equivalently internalized EHEC cultured with and without bilirubin (P-value>0.05) (Fig. 3A). Interestingly, EHEC treated with bilirubin showed several-fold higher levels of live bacteria than the untreated control, suggesting bilirubin reduced the rates of killing by J774A.1 macrophages (Fig. 3B). These data remained consistent between experiments when macrophages were primed or unprimed with LPS (Fig. S4A and B). Consistent with data from Figure 2, biliverdin failed to promote survival of EHEC within macrophages, even when supplemented into media during initial exposure (Fig. 3B). Furthermore, the known lipophilic antioxidant vitamin E (α-tocopherol) led to an increased survival of EHEC within macrophages, although at a reduced level compared to bilirubin (Fig. 3B) [22]. These data strongly support the notion that bilirubin can act as a lipophilic antioxidant capable of protecting EHEC from exogenous and host (macrophage)-generated ROS.

### Bilirubin is toxic to several tested Gram-positive pathogenic bacteria

**Figure 4:**
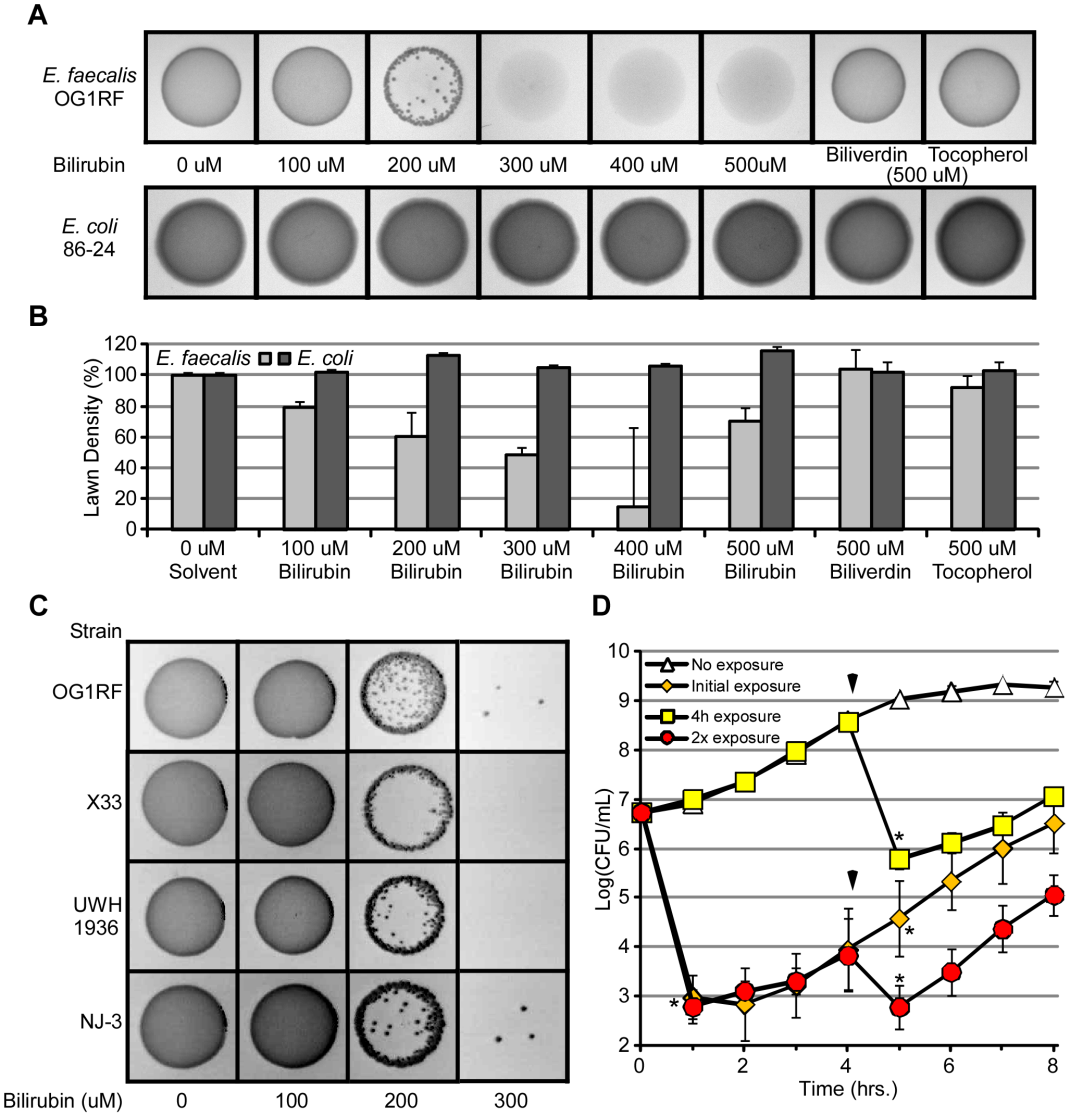
Bilirubin decreases the viability of *E. faecalis*. (A) An equal number of CFUs of *E. faecalis* or *E. coli* (O157:H7 86-24) were mixed with increasing amounts of bilirubin (0, 100, 200, 300, 400, and 500 µM), biliverdin (500 µM), or α-tocopherol (500 µM), before spotting onto LB agar. Plates were grown at 37°C overnight. (B) Densitometry of bacterial lawns was measured using ImageJ software. (C) *E. faecalis* strains OG1RF (oral origin), X33 (fecal origin), UWH 1936 (blood origin), and NJ-3 (peritoneal fluid origin) were exposed to increasing concentrations of bilirubin (100, 200, or 300 µM) prior to spotting onto an LB-agar plate. Bacterial growth was captured by imaging the plates, and images were modified by adjusting brightness and contrast to best display colony formation (darker areas of image). (D) Growth of *E. faecalis* with bilirubin (yellow squares) or without bilirubin (white diamonds) was quantified by CFU plating. Bilirubin was titrated into *E. faecalis* cultures initially with bilirubin (red squares) and initially without bilirubin (orange squares) after 4 hours (marked by arrows). Error bars represent ± one standard deviation, n = 3, and (*) denotes a significant (P≤0.05) difference between treated samples and solvent-treated samples.

The finding that bilirubin could promote the survival of EHEC when exposed to ROS prompted us to assess if other bacteria, particularly Gram-positives, might also benefit by interacting with bilirubin. To test this hypothesis, we determined the effect of bile pigments on *Enterococci*, which are similar to *E. coli* in that they are part of the aerobic flora within the large intestine [23]. For this purpose, we used *E. faecalis*, an opportunistic enteric pathogen, and otherwise commensal organism, which has wide-spread antibiotic resistance [17], [24]. Increasing the concentration of bilirubin led to a decrease in the numbers of *E. faecalis*, as judged by bacterial lawn formation on agar plates and quantified by densitometry (Fig. 4A and B). Consistent with the trends already noted with EHEC, no effect was observed when the experiment was repeated with biliverdin or α-tocopherol, even at very high concentrations (Fig. 4A and B). Furthermore, three other strains of *E. faecalis* with different origins of isolation (feces, blood, or peritoneal fluid) were tested against bilirubin by this method. Each strain responded similarly to OG1RF, with substantial decreases in viable cells as bilirubin increased in concentration (Fig. 4C). These data suggest, in contrast to *E. coli*, that bilirubin exerts a toxic effect on *E. faecalis*, and which is seemingly independent of strain and origin.

To provide a greater understanding into how bilirubin may be affecting *E. faecalis*, we monitored changes in colony forming units over time during planktonic growth while exposed to bilirubin. Exposure to bilirubin reduced the amount of viable bacteria by approximately 4 orders of magnitude within a single hour (Fig. 4D, compare white triangles to orange diamonds), suggesting that bilirubin had a direct and immediate effect on cell viability and survival. Interestingly, given enough time, the remaining cells started to slowly grow and nearly reached the initial inoculum by 8 hours. To determine if this observation could be due to an intrinsic adaptation of *E. faecalis* to bilirubin as opposed to a change in the physical properties of the pigment, the experiment was repeated but with a secondary addition of bilirubin to growing cultures at 4 hours (Fig. 4D, see arrows, yellow squares and red circles). *E. faecalis* that was previously exposed to bilirubin decreased in viable CFUs after the new infusion, suggesting the new population was not intrinsically resistant (i.e. underwent a mutational event that conferred resistance) but rather that the bilirubin was either titrated out or lost activity with time. This data further supports the idea that bilirubin contains an intrinsic ability to disrupt cell viability.

Finally, to determine if this effect was specific to *E. faecalis* as opposed to a more general effect on bacterial cells with a single membrane, we determined the effect of bilirubin on two medically-significant Gram-positive pathogens, *Bacillus cereus* and *Staphylococcus aureus*. As observed in Table 1, bilirubin also led to a dramatic decrease in cell viability of these two bacteria without affecting the control, *E. coli*. When taken together, these additional results suggest bilirubin is highly toxic to three Gram-positive bacterial species, including *E. faecalis*, and likely works through a direct, rapid, and physical mechanism of disruption.

**Table 1:** Fold reduction in viability of bacteria exposed to bilirubin.

Bacteria	Strain	Fold Reduction	% Std. Dev.
*E. coli*	86-24	1.0	17%
*E. faecalis*	OG1RF	610,000	54%
*B. cereus*	1048	131,111	43%
*S. aureus*	MW2	57	104%

Mid-log phase bacteria were exposed to 200 µM bilirubin and plated to determine CFUs. Fold reduction was calculated by the quantity of bacteria unexposed to bilirubin divided by the quantity present when exposed to bilirubin, n = 3.

### Bilirubin destabilizes the membrane of Gram-positive bacteria

The finding that bilirubin was cytotoxic to *E. faecalis* and that this effect could be titrated out promoted us to hypothesize that unconjugated bilirubin was, by virtue of its lipophilic properties, intercalating into the bacterial membrane and causing disruption of membrane function. Bile salts, amphiphilic detergents capable of disaggregating lipids, are used for selection and enrichment of Gram-negative bacteria, through a similar mechanism [25]. Investigation into bile resistance suggests the outer membrane of Gram-negative bacteria slow the diffusion of bile salts into the inner membrane, and mechanisms of efflux and enzymatic alteration lead to higher levels of resistance when compared to Gram-positive bacteria, yet many of the mechanisms of bile salt resistance remain undetermined. Bile salts may increase membrane permeability or instability, potentially leading to lysis, in both bacteria and erythrocytes [25].

**Figure 5:**
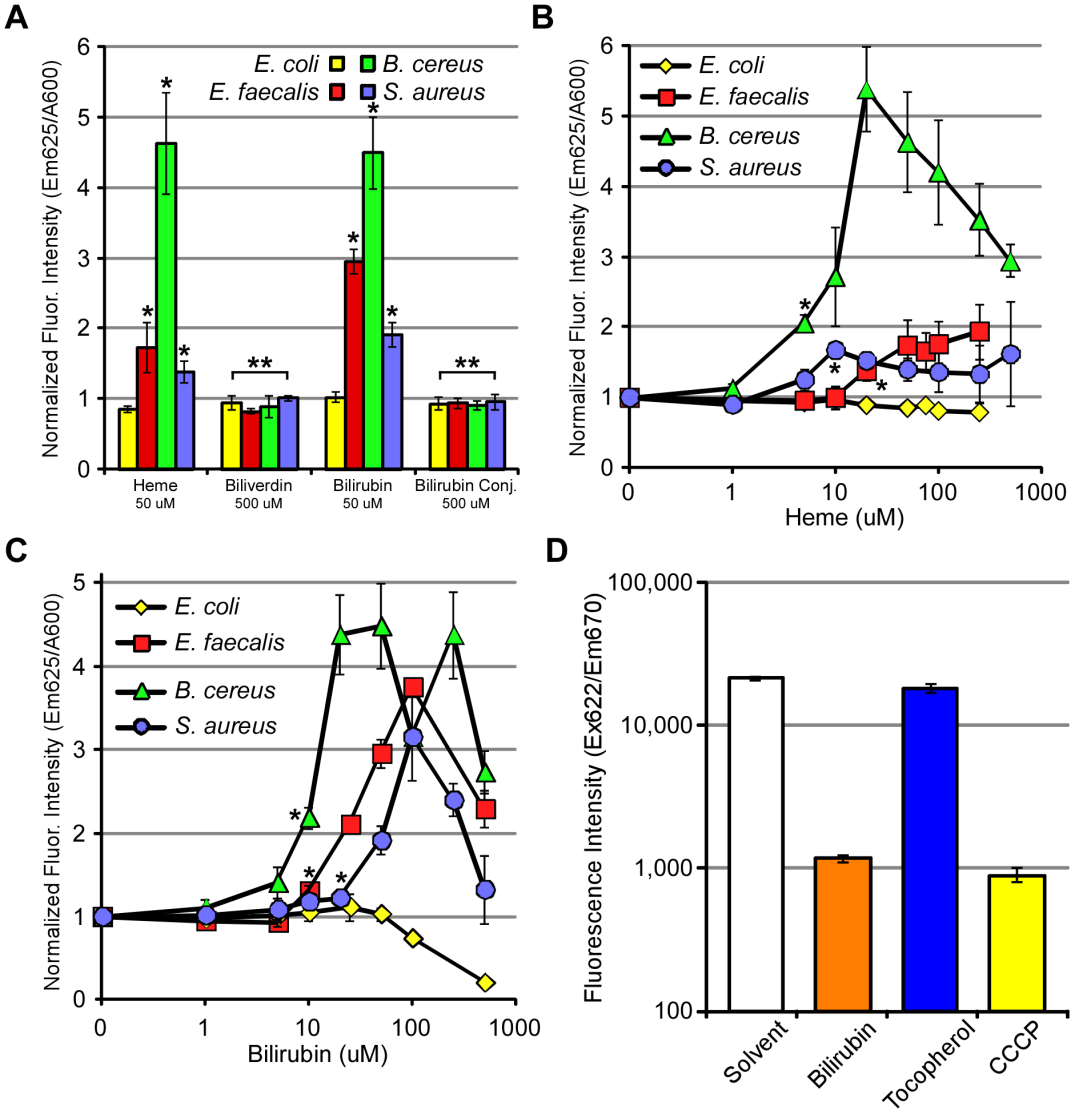
Membrane disruption by bilirubin. (A) Bacteria including EHEC (86-24), *E. faecalis*, *S. aureus*, and *B. cereus* were incubated with heme, biliverdin, bilirubin, and bilirubin ditaurate (0, 1, 5, 10, 20, 50, 75, 100, 250 and 500 µM for heme and bilirubin (B,C), or 500 µM for biliverdin and bilirubin ditaurate (A)) and membrane permeability monitored by propidium iodide fluorescence. (D) *E. faecalis* OG1RF was cultured to mid-log phase and exposed to bilirubin, alpha-tocopherol, and CCCP (each 100 µM). DiSC_3_(5) (1 µM final concentration), a fluorescent compound which increases in intensity when associated with polarized membranes, was supplemented into cultures before quantifying the fluorescence intensity at excitation 622 nm and emission 670 nm. Error bars represent ± one standard deviation, n = 3, and (*) denotes a significant (P≤0.05) difference while (**) denotes a non-significant difference (P>0.05) between treated samples and solvent-treated samples.

We therefore tested if bilirubin exposure to Gram-positive bacteria caused membrane instability by adding propidium iodide to EHEC and *E. faecalis* previously incubated with increasing concentrations of heme, biliverdin, bilirubin, and bilirubin ditaurate. Propidium iodide (MW = 668) fluoresces intensely when associated with DNA, and its use in this context would indicate if the membrane became permeable after bile pigment exposure [26]. As expected, significant increases in fluorescent intensity occurred in heme-treated *E. faecalis*, as compared to solvent-treated samples (Fig. 5A and B). It is thought that heme toxicity may be partially related to its ability to intercalate in membranes [1]. Interestingly, bilirubin-treated *E. faecalis* also showed an increase in propidium iodide-specific fluorescence compared to solvent-treated cells (Fig. 5A and C). However, this was not true of *E. faecalis* treated with biliverdin or bilirubin ditaurate, which showed no increase in fluorescence. Both *B. cereus* and *S. aureus* also showed increases in propidium iodide fluorescence upon exposure to bilirubin, suggesting the membranes of Gram-positive bacteria are permeabilized by unconjugated bilirubin (Fig. 5A and C). This hypothesis was further tested with the use of DiSC_3_(5), a probe that binds polarized membranes, yielding fluorescence [27]. The exposure of *E. faecalis* to the control conditions, solvent or α-tocopherol, yielded strong fluorescence, consistent with intact polarized membranes (Fig. 5D, open and blue bars). In contrast, bilirubin treatment led to more than a two-fold decrease in the fluorescence, which was similar to treatment with the proton gradient uncoupler, CCCP, a known de-polarizing agent (Fig. 5D, orange and yellow bars). Taken together, the decrease in fluorescence induced by bilirubin is consistent with a decrease in membrane polarization, suggesting bilirubin disrupts membrane physiology, presumably via membrane permeabilization.

### Membrane permeability increases leads to a decrease in the respiratory metabolism

**Figure 6:**
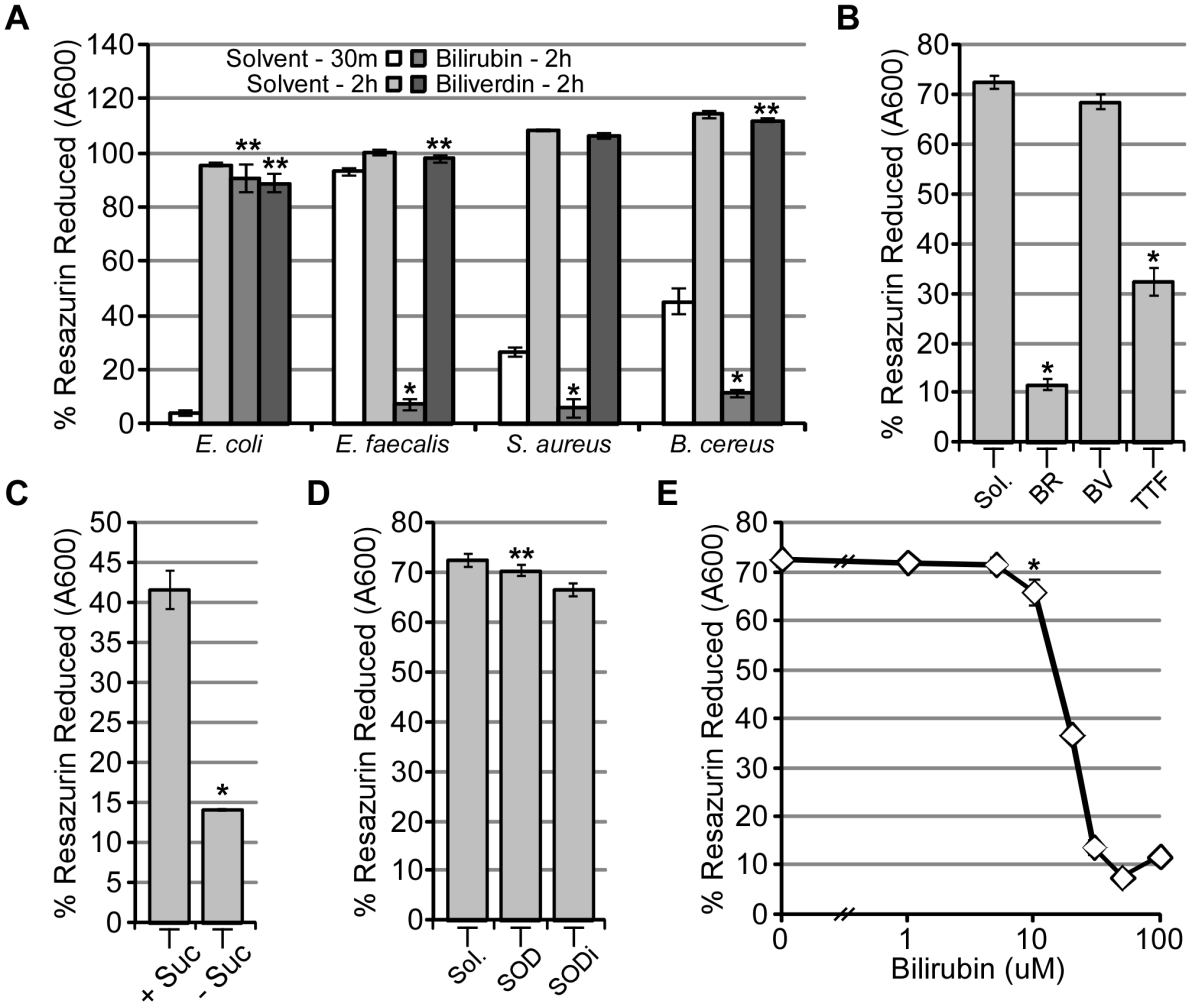
*E. faecalis* metabolism decreases after exposure to bilirubin. (A) *E. coli* (86-24), *E. faecalis*, *B. cereus*, and *S. aureus* were supplemented with resazurin and incubated with heme (50 µM), biliverdin (500 µM), bilirubin (50 µM), and bilirubin ditaurate (500 µM) for either 30 minutes or 2 hours. Unreduced resazurin was monitored by absorbance at 600 nm. (B) *E. faecalis* cultures were supplemented with resazurin and solvent (NaOH, Sol.), bilirubin (100 µM, BR), biliverdin (100 µM, BV), or TTF (1 mM, a known inhibitor of succinate dehydrogenase) while incubated in 1× PBS with 0.5% sucrose for 30 minutes at 37°C. (C) Similar to panel B, *E. faecalis* cultures were supplemented with resazurin and either superoxide dismutase (SOD, 1000 U/mL) or heat-inactivated superoxide dismutase (SODi, 1000 U/mL). (D) *E. faecalis* supplemented with resazurin and diluted into 1× PBS with 0.5% sucrose (+Suc.) or without sucrose (−Suc.) and incubated for 30 minutes at 37°C. (E) *E. faecalis* supplemented with resazurin and increasing amounts of bilirubin (1, 5, 10, 20, 30, 50, and 100 µM) in similar conditions as panels B and C. Error bars represent ± one standard deviation, n = 3, and the (*) denotes a significant (P≤0.05) difference while (**) denotes a non-significant difference (P>0.05) between treated samples and solvent-treated samples.

We hypothesized that bilirubin-mediated membrane instability would decrease the respiratory metabolism at the cell membrane, an essential cellular mechanism which can be measured with high sensitivity. This in turn could explain the substantial cytotoxic effect bilirubin has on *E. faecalis*, *S. aureus*, and *B. cereus*. Assays to quantify bacterial and eukaryotic respiratory metabolism have been developed and principally use artificial electron acceptors, such as tetrazolium salts and resazurin based compounds, which change spectral absorbance after cellular metabolic reduction. In this regard, we used resazurin to monitor the metabolic activity in *E. faecalis* since these bacteria are highly active at reducing artificial electron acceptors compared to other bacteria (Fig. 6A) [28]. Using this system, we observed that the reduction of resazurin is dependent on the electron transport system, as demonstrated by a decrease in resazurin reduction when *E. faecalis* is incubated with the succinate dehydrogenase inhibitor TTF (Fig. 6B). In addition, the abundance of a carbon source is also essential for resazurin reduction, since a decrease in the reduction of resazurin was observed when sucrose is removed from the reaction buffer (Fig. 6C). These controls aside, we hypothesized the reduction of resazurin would be increased by the extracellular production of superoxide, a characteristic of *E. faecalis*. Production of extracellular superoxide requires the autoxidation of molecular oxygen by demethylmenaquinone, a membrane associated quinone reduced by cellular dehydrogenases [16]. Indeed, cultures supplemented with both resazurin and superoxide dismutase (SOD) resulted in an equivalent reduction of resazurin within each culture (Fig. 6D), thereby demonstrating that resazurin reduction must occur at the cellular membrane since resazurin was not reduced by extracellular superoxide. Hypothesizing that those concentrations of bilirubin that increase the membrane permeability of *E. faecalis* would similarly decrease the amount of resazurin reduced, we tested resazurin reduction in the presence of bilirubin and *E. faecalis*. As demonstrated in Fig. 6E, as little as 10 µM bilirubin led to a decrease in the reduction of resazurin, which incidentally is the same concentration of bilirubin which increases membrane permeability of *E. faecalis* (Fig. 6C). Since these results suggest bilirubin inhibits cellular metabolism at concentrations that increase the cellular permeability of *E. faecalis*, we conclude that bilirubin-mediated cytotoxicity towards *E. faecalis* likely stems from the physical disruption of membrane permeability, thereby perturbing membrane polarity and aerobic respiration.

## Discussion

Earlier work to characterize bilirubin and other bile pigments, such as those conducted by Stocker *et* al., demonstrated the antioxidant potential of these small molecules [1], [10]. Lipid-soluble bilirubin effectively scavenged peroxyl radicals both in solution and within liposomes *in vitro* and more so than α-tocopherol, one of the best known antioxidants. Both bilirubin and biliverdin were capable of interacting synergistically with α-tocopherol, acting as chain-breaking antioxidants inhibiting lipid-peroxidation. *In vivo*, antioxidant models of bilirubin indicate biliverdin reductase, the enzyme responsible for creating bilirubin from biliverdin, interacts with bilirubin upon its oxidation to biliverdin, effectively removing a free radical in circulation and regenerating the cellular pool of bilirubin [29]. Since most species of bacteria (excluding cyanobacteria) lack biliverdin reductase activity, the cyclical antioxidant process of biliverdin and bilirubin and back would not likely occur within bacteria, though significant amounts of bilirubin are supplemented into the human intestine daily [30], [31].

Concentrations of bilirubin within the human body can range dramatically depending on the physiologic location and individual's health. Healthy adult serum bilirubin concentrations are nearly 20 µM, yet can reach concentrations higher than 300 µM during hyperbilirubinemia [1], [32]. Bilirubin is found in conjugated forms at millimolar concentrations in bile, yet deconjugation occurs through enzymatic and non-enzymatic processes, leading to unconjugated bilirubin in bile isolated from human gall-bladders ranging from 50 to 150 µM, well above the antioxidant potency concentrations observed in human serum [33], [34], [35]. Human bile contains β-glucuronidase activity, an enzyme responsible for deconjugating bilirubin from glucuronic acid [7]. The effects of bilirubin observed in this study are within the range of bilirubin concentrations observed at various locations in the body, especially the intestine.

We sought to determine if bilirubin could function as an antioxidant for GI-associated bacteria. Little is known about the interaction of this metabolite with commensal or pathogenic bacteria and a bioactive role for bilirubin would be expected to influence bacterial homeostasis in the gut. Using characterized ROS-generating toxin, plumbagin, and a photosensitizing agent, Rose Bengal, with a well-known GI pathogen (EHEC), we developed assays to test the antioxidant potential of compounds with pathogenic bacteria [18], [21]. These assays allowed us to determine that bilirubin can be a functional antioxidant and protect EHEC from oxygen radicals. Since the bile pigments biliverdin, which has a very similar structure to bilirubin but does not have the capacity to accept free electrons, and bilirubin ditaurate, which can accept free electrons but is soluble, do not protect EHEC from ROS, the data suggest that the mechanism of protection is through the direct neutralization of free radicals after bilirubin associates with the bacterial cell. Previous work has demonstrated rapid cellular uptake of free bilirubin in eukaryotic cells [36]. Indeed, we have observed that all of the bilirubin given to these cells in culture either ends up associated with the bacteria (∼30%, being in the membrane, periplasm, and/or cytosol) or the insoluble pellet after clarification of the lysate (∼70%, Fig. S2). Furthermore, expression of BVR in EHEC that were fed biliverdin did not lead to protection from ROS. Although it is possible not enough bilirubin was made in this reaction, it is tempting to speculate that bilirubin's association with membranes after exogenous addition serves to protect from extracellular ROS generated at the bacterial surface by the action of plumbagin (*i.e.* an “outside in” rather than an “inside out” mechanism of protection). Such a mechanism would protect from radical-mediated lipid peroxidation, a known and potent ROS-generated effect on cells, by possibly neutralizing ROS at membrane [10]. The finding that macrophages, which use oxygen radicals to destroy engulfed pathogens, are less efficient at killing bacteria pre-loaded with bilirubin indicates this effect may be biologically relevant, perhaps mitigating host ROS during the infectious process.

Between the rescue of EHEC from ROS-induced growth impairment and the decreased phagocytic killing of EHEC within macrophages, the evidence that bilirubin can act as a potent antioxidant at physiologically relevant concentrations for EHEC appears strong. In addition to what appears to be a direct physical interaction with bacteria, bilirubin may also alter host gene expression. Indeed, a preliminary screen of proteomic changes that occur in EHEC upon exposure to bilirubin yielded several changes (Fig. S5 and S6, Table S1). 2D-DIGE analysis identified more than 50 spots modified in abundance upon exposure to bilirubin. After identifying ten proteins found in higher or lower abundance (though this could not be confirmed at the transcriptional level – Fig. S6), we found four redox enzymes involved in cellular redox related metabolism. In a previous study by Shao *et* al., *Helicobacter pylori* exposed to bile increased the abundance of several metabolically-related redox enzymes. Little investigation was reported as to the reason or mechanism behind the increased abundance other than the hypothesis that the enzymes could possibly regulate bacterial homeostasis after bile exposure [37]. Previous research supports toxic effects of bilirubin leading to uncoupling of oxidative phosphorylation, inhibition of hydrolytic enzymes, dehydrogenases, and enzymes involved in the electron transport system [1], [38]. Possibly, several redox enzymes increase in abundance after bilirubin exposure to compensate for inhibitory effects, or alternatively, bilirubin may act as a signal to prime the bacterial cells to prepare for host-generated ROS. Given that the products of these genes showed an increase upon analysis of their proteins levels after exposure to bilirubin, but not at the transcriptional level, this data suggests the effects of bilirubin on these proteins is post-transcriptional. This might occur in a number of ways, including that bilirubin may enhance the rates of translation of these genes, or act to stabilize them once made.

Interestingly, we observed bilirubin is toxic to the human commensal *E. faecalis*. At concentrations of bilirubin found to protect EHEC from ROS cytotoxicity, *E. faecalis* was readily killed. This was also true of at least two other distinct Gram-positive species. Interestingly, biliverdin did not cause a change in the growth of *E. faecalis* on agar, demonstrating specificity toward bilirubin activity. Furthermore, viability decreases dampened with time, but could be restored with fresh addition of bilirubin. Bilirubin increased the propidium iodide uptake by cells, and caused a disruption of the membrane potential. When taken together, this information led us to conclude that bilirubin decreases the viability of the assayed species of Gram-positive bacteria through direct intercalation into the bacterial plasma membrane, further disrupting essential cellular functions. This is similar to the effects of bile salts and sodium dodecyl sulfate on bacterial cells and argues bile contains several chemically distinct compounds that act on bacterial membrane structures [25], [39]. Since this susceptibility to bilirubin is not observed with the four distinct strains of *E. coli* we tested, it is tempting to speculate that Gram-negative bacteria resist the membrane intercalating effects of bilirubin because they contain two membranes (inner and outer), with the outer absorbing most of the molecule, sparing the inner from damage. In this regard, having an outmost layer of membrane that contains a ROS neutralizing molecule, can, under the certain conditions, actually provide a benefit. This model would support that since Gram-positive bacteria contain a single membrane, their susceptibility may be due to bilirubin disrupting this single structure, which serves the same functions as the two membranes of Gram-negative bacteria. In this model, structural differences in the surface of Gram-negative and Gram-positive bacteria may be the determinant as to whether bilirubin is beneficial or harmful. It is also possible that the structure or composition of the outer membrane in Gram-negative bacteria confers resistance to the intercalating effects of bilirubin, as compared to Gram-positive bacteria. For example, changes in the structure of the O-antigen of Salmonella changes the susceptibility of this bacterium to bile acids [40]. Future studies will be needed to address this exact mechanism.

Most work on bilirubin has ignored its potential role in modulating intestinal biology, with research largely focused on bilirubin-associated toxicity and metabolism relating to neurological damage during severe hyperbilirubinemia [8], [36], [38], [41]. Bilirubin at high concentrations can enter the central nervous system and cause acute encephalopathy [1], [32]. Lipid-soluble bilirubin associates quite rapidly with exposed cells, which correlates with an increase in cell death [3], [36]. Bilirubin may act as an uncoupler of oxidative phosphorylation and an inhibitor of respiration [38], [42], and also leads to an increase in the loss of cellular proteins [36]. Taken together, these data suggest bilirubin toxicity may be related to its ability to disrupt membrane integrity in higher organisms and data presented here with bacteria provide experimental support for these ideas [1], [25].

Bile salts lead to the selection and enrichment of Gram-negative bacteria species, presumably through negative effects on surface membranes [25]. Likewise, heme toxicity may include membrane destabilization [1]. Our data suggests the plasma membranes of *E. faecalis* become permeabilized above concentrations of bilirubin as low as 10 µM, an observation which is shared with heme exposure. This was also true of *B. cereus* and *S. aureus*, which suggests bilirubin-induced membrane permeability may be a universal property common to all Gram-positive bacteria.

What are the consequences of bilirubin's action on Gram-positive bacteria? To correlate decreases in bacterial viability with increased membrane permeability, we investigated the changes in overall metabolic activity of *E. faecalis* when exposed to bilirubin. Metabolic activity was quantified using artificial electron acceptor resazurin [28]. As expected, exposure to bilirubin decreased the rate at which *E. faecalis* reduced resazurin at similar concentrations which previously increased permeability of membranes. If these results are extrapolated to other Gram-positives (indeed, in this report we present consistent data that supports this extension), it is possible that bilirubin acts as an antagonist against some bacterial species in the diverse GI tract, a process that may influence the abundance and composition of the GI flora. Unlike bile salts which can be prominently reabsorbed during transit down the GI tract, unconjugated bilirubin has been shown to increase in comparison to gallbladder concentrations as one moves through the intestine [9], [43], [44]. β-glucuronidases are well known to be secreted with bile and facilitate the reverse reaction of conjugation performed by the liver to conjugate bilirubin to either one or two glucuronic acid molecules [7]. Though a physiologic function for bile-secreted β-glucuronidases is not well described, secreted enzymes may function to increase the concentration of unconjugated bilirubin during bile transit down the GI tract by utilizing the high concentration of conjugated bilirubins. Further, the concentration of β-glucuronidase can be increased by populations of coliform bacteria and to a much greater extent by *Clostridia* species, both of which actively secrete β-glucuronidases, implicating these bacteria in initial bilirubin breakdown within the GI tract [6], [45]. Intestinal epithelia expression of UGT1A1, the enzyme responsible for biotransformation of bilirubin by conjugating the molecule with glucuronic acid, decreases from the ileum to the cecum, suggesting a driving force toward conjugated bilirubin diminishes as intestinal contents approach the colon [46]. Thus, it is possible that bilirubin exerts differential effects on different communities as it traverses down the intestine, being modified along the way by native species.

This information, in combination with data presented here, allows us to propose the hypothesis that bilirubin is simultaneously acting as an antioxidant and membrane-destabilizing agent within the GI tract. This proposed dual function may impose a selective pressure on resident and pathogenic bacteria, allowing bacteria capable of resisting the toxic effects of bilirubin to utilize the antioxidant properties for protection from ROS. In this study we demonstrated both activities of bilirubin with various intestinal-associated bacteria, suggesting the function of bilirubin does not stop until it passes from the GI tract. Future work will define bacterial responses towards bilirubin, determine their relationship to oxidative stress, and be correlated to possible responses observed in the intestinal tract. It will be necessary to test these ideas against a greater number of bacterial species and in relevant models of animal infection. Animal models of infection that center on intestinal bilirubin and allow one to probe these questions, although non-existent, will need to be developed to answer these questions. Such models may include the use of inhibitors or surgery to inhibit bile (and thus bilirubin) secretion into the intestine, or the use of knockout (e.g Nox or Duox-deficient) mice to further explore certain host components that generate ROS.

## Methods and Materials

### Bacterial strains and reagents

Bacterial strains used in this study included *Escherichia coli* serotype O157:H7 (EHEC) strain EDL933 (ATCC# 700927) and 86-24 [47], [48], *E. coli* serotype O104:H4 2011 German outbreak (EAEC) (generously provided by Dr. Alison Obrien, Uniformed Health Services), *Enterococcus faecalis* strain OG1RF (ATCC# 47077) [24], *E. faecalis* strain X33 (ATCC# 27274), *E. faecalis* strain UWH 1936 (ATCC# 49533), *E. faecalis* strain NJ-3 (ATCC# 51299), *Staphylococcus aureus* strain MW2 (ATCC# BAA-1707), and *Bacillus cereus* strain NRS 248 (ATCC# 10987). “Commensal” *E. coli* strains (CN-5 and CN-7) were isolated from healthy donor stool samples and identified by selection on MacConkey agar plates, Gram-staining, and by VITEK analysis (generously conducted by Dr. Audrey Wanger, University of Texas Health Science Center). *E. coli* strains were grown at 37°C in Luria-broth (LB), while *E. faecalis*, *S. aureus*, and *B. cereus* were grown at 37°C in brain heart infusion broth (BHI). Cultures were started from a single colony selected from LB or BHI agar plates using aseptic techniques. Kanamycin (25 ug/mL) was supplemented to media for the selection of EHEC (86-24).

Whole ox bile was purchased from Fluka Analytical (B3883-25G); rabbit bile from Pel-Freez Biologicals (41206-1); hemin (heme) from Sigma Life Science (H9039-100G); biliverdin hydrochloride (biliverdin) from Frontier Scientific (B655-9); unconjugated bilirubin (bilirubin) from Alfa Aesar (A17522); bilirubin ditaurate from Frontier Scientific (B850); plumbagin from Sigma Life Science (P7262-100MG); α-tocopherol from Alfa Aesar (10191-41-0); bovine serum albumin (BSA) from Fisher Scientific (9048-46-8); resazurin from Acros Organics (62758-13-8); MBTH from Research Organics (0133M); kanamycin from EMD (OmniPur 25389-94-0); ampicillin from USB corporation (69-52-3); spectinomycin from MP Biomedicals, LLC (158993); Rose Bengal Sodium Salt from Santa Cruz Biotechnology, Inc. (sc-203757); 3,3′-Dipropylthiadicarbocyanine iodide (DiSC_3_(5)) from Santa Cruz Biotechnology, Inc. (sc-209690). Human bile was generously provided by Dr. Mary Estes (Baylor College of Medicine). Minimal media IDM was formulated as described in [49]. Reagents were solubilized as follows: ox bile, heme, biliverdin, bilirubin, bilirubin ditaurate, and BSA in 0.1 M NaOH; α-tocopherol in 100% ethanol, followed by a 1 to 100 dilution for stock concentrations in 0.1 M NaOH; Pbm and DiSC_3_(5) in DMSO; and Rose Bengal Sodium Salt in sterile milliQ water.

Expression of biliverdin reductase in EHEC 86-24 was conducted through the use of a pUC19 vector system. Briefly, *bvr* was amplified from ATCC #MGC-14706 using the forward primer (GATCGATCGTCGACTATGAATGCAGAGCCCGAG) and reverse primer (GTAATGGGTACCTTATTATGCATAATCCGGAACATCATACGGATACTTCCTTGAACAGCAATATTTCTG) followed by restriction enzyme digesting with both SalI and KpnI enzymes before ligating with SalI and KpnI digested pUC19 vector (Invitrogen, #54357). Ligation products were heat shock transformed into NEB 5 alpha competent *E. coli* (NEB, #C2987I) and positive colonies were selected by blue white screening. Positive colonies were further screened by PCR, restriction enzyme digest, and finally by DNA sequencing. Correctly cloned pUC19-BVR was transformed by electroporation into EHEC 86-24 and selected for on ampicillin LB-agar plates. EHEC 86-24 pUC19-BVR was determined to express BVR by anti-HA Western blot against the HA-tag incorporated into the C-terminus of BVR.

### Bacterial growth and viability

EHEC (EDL933 and 86-24) were inoculated into cultures at a final OD_600_ of 0.03 (1 cm pathlength, Beckman Coulter DU 800) with or without plumbagin, whole bile (ox, rabbit, or human), biliverdin, bilirubin, bilirubin ditaurate, BSA, and α-tocopherol at concentrations indicated in the figures at 37°C with shaking. For experiments including EHEC 86-24 with pUC19 or pUC19-BVR, media was further supplemented with ampicillin (100 µg/mL, final concentration). Growth was monitored using a Bioscreen C machine, with wideband absorbance from 420–580 nm. Time to mid-log phase was calculated through linear interpolation of data points that were at mid-log densities. Monitoring bacterial quantities (CFU/mL) by optical density has been demonstrated in our laboratory as having a strong correlation (data not shown). For cultures supplemented with Rose Bengal, bacteria prepared in the same fashion as above were supplemented with Rose Bengal (final concentration 750 uM) and grown in 1 mL cultures under fluorescent lighting for 12–14 hours. Bacterial growth density was quantified using a Tecan Infinite M200 Pro plate reader to measure the absorbance at 600 nm. For the experiments in Figure 4, bacterial lawn formation was monitored by mixing mid-log phase bacteria with bilirubin or biliverdin in PBS at an OD_600_ value of 0.5. Mixtures were then diluted 1∶1000, of which 10 µL were spotted onto a LB agar plate. Plates were incubated at 37°C overnight and imaged using either a transilluminator (W/M-26XV) in a UVP BioSpectrum 810 Imaging System or UVP Benchtop Imaging System. Densitometry of agar plates was calculated using the freeware program ImageJ (version 1.45s, Wayne Rasband, NIH, USA). Colony forming unit (CFU) determination was conducted by serial dilution of cultures and spotting on LB agar plates, grown overnight at 37°C.

### Macrophage killing assays

Murine macrophages (J774A.1, ATCC# TIB67) were cultured at 37°C in 5% CO_2_ in RPMI-1640 with L-glutamine (Lonza 12-702F; RPMI) supplemented with 100 µg/mL spectinomycin and ampicillin and 10% heat-inactivated fetal bovine serum (JR Scientific, Inc.; 43635). Intercellular amounts of bacteria were determined in a method similar to [50]. Briefly, EHEC 86-24 was cultured for 4.5 hours in the presence of biliverdin, bilirubin, or α-tocopherol (all 250 µM), washed extensively with 1× PBS, diluted in RPMI, and added to J774A.1 cultures at an MOI of 3. Bacteria were incubated with macrophages for 30 minutes prior to removal and macrophages were washed with 1× PBS to remove unbound bacteria. Growth media containing antibiotics was added to washed macrophages to kill bacteria not phagocytosed. To lyse the macrophages, media was removed and cells washed with 1× PBS before addition of milliQ water. Samples were then serially diluted and plated to determine CFU per well.

### Analysis of cell permeability and membrane functionality

Mid-log phase bacteria cultured in LB were incubated with heme, biliverdin, bilirubin, and bilirubin ditaurate in milliQ water at an OD_600_ of 0.4 for 30 minutes at 37°C, pelleted by centrixfugation, and washed once with 1× PBS. Propidium iodide (10 µM, Invitrogen; B34954) was added to cells and incubated at 25°C for 10 minutes in the dark, followed by analysis at excitation and emission wavelengths of 535 and 625 nm, respectively. Similarly, bacteria prepared in by the same method were supplemented with DiSC_3_(5) (final concentration 1 uM) and analyzed using 622 nm excitation and 670 nm emission wavelengths for fluorescent quantification. Fluorescent quantification was conducted using a Tecan Infinite M200 Pro fluorescent plate reader.

### Resazurin reduction

Mid-log phase bacteria were diluted to an OD_600_ of 0.1 in minimal media or 1× PBS with 0.5% sucrose and supplemented with resazurin (50 µM) and either heme (50 µM), biliverdin, bilirubin, bilirubin ditaurate, TTF, superoxide dismutase, and heat-inactivated superoxide dismutase. These cultures were incubated for 30 minutes to 2 hours at 37°C while shaking. After allotted time, cultures were spectrophotometrically measured using a Tecan Infinite M200 Pro for absorbance at 600 nm wavelength. As unreduced resazurin has a strong absorbance at 600 nm, a property which diminishes as the compound is reduced, measurements were taken at A600 and background was subtracted from cultures not supplemented with resazurin. Using reactions not containing reducing agents and those not containing resazurin, the amount of resazurin reduced in each culture could be calculated on a percentage basis.

### Two-dimensional Difference Gel Electrophoresis (2D-DIGE) and qRT-PCR

2D-DIGE was used to investigate the proteomic response of *E. coli* to bilirubin and was performed by Applied Biomics, Inc. (Hayward, CA). Mid-log phase *E. coli* str. 86-24 was exposed to bilirubin, biliverdin (250 µM), or a solvent control (NaOH) for 4.5 hours, bacteria were washed four times with ice cold PBS, and then flash frozen with dry ice and ethanol. Protein from samples was extracted using 2-D lysis buffer (7 M urea, 2 M thiourea, 4% 3-((3-cholamidopropyl)-dimethylammonio)-1-propanesulfonate (CHAPS), 30 mM Tris-HCl, pH 8.8) and quantified. Equivalent amounts of samples were covalently labeled with CyDye and run on first dimension isoelectric focusing, followed by second dimension SDS-PAGE. Image analysis was conducted with DeCyder software. Prominently changed spots were chosen for analysis by mass spectrometry (MALDI/TOF/TOF) and received data was used in peptide fingerprinting for protein identification. Bacteria prepared in the same fashion were subjected to RNA isolation using the Qiagen RNEasy Mini kit (Cat. no. 74104). Quantitative reverse-transcription PCR was conducted using the Qiagen QuantiFast SYBR Green RT-PCR kit (Cat. no. 204154) with an Applied Biosystems 7500 Real-time PCR system. Analysis of data was conducted without using an efficiency correction as each reaction was observed to have efficiencies of greater than 90%.

### Statistical analysis

Solvent and treated samples were compared using the students T-test to determine significance. Values were considered statistically different if the comparison of their groups yielded a p-value less than 0.05.

## Supporting Information

Figure S1 **Chemical structure of heme catabolites.** Heme is oxidatively cleaved by heme oxygenase (Hmox) to form ferrous iron (Fe^II^), carbon monoxide (CO), and biliverdin. Biliverdin is further reduced by biliverdin reductase (BVR) to form the product bilirubin. Glucuronic acid molecules are conjugated to bilirubin by the UGT1A1 enzyme (expressed highly in the liver), forming bilirubin di-glucuronide, which can be easily passaged into bile [4]. Proposed reactive hydrogen(s) for neutralizing ROS though hydrogen donation are annotated within the dotted circles for each structure [10].
(TIF)

Figure S2 **Association of bilirubin with EHEC upon exposure.** EHEC strain 86-24 was incubated with or without bilirubin (approximately 100 µM) for 30 minutes, the cells washed twice with PBS prior to resuspention in ice cold milliQ water, and then sonicated for 12 seconds. Supernatants were collected prior to incubation (Pre-inc.), after incubation (Post-inc.), after the 1^st^ wash (Wash), after the 2^nd^ wash (not shown), and after lysis (Lysate). Bilirubin was quantified as described in Nagaraja *et* al. [19]. Error bars represent ± one standard deviation, n = 3, and (*) denotes a significant (P≤0.05) difference between EHEC and bilirubin samples and bilirubin alone samples.
(TIF)

Figure S3 **Expression of human BVR does not rescue EHEC from plumbagin-induced growth inhibition.** (A) EHEC strain 86-24 containing the pUC19-BVR (pBVR) plasmid and EHEC strain 86-24 containing pUC19 were cultured with plumbagin (50 µM) to induce growth inhibition. Cultures were supplemented with biliverdin (500 µM, green bars) or bilirubin (500 µM, yellow bars) or solvent (NaOH, white bars). The time to mid-log phase was calculated from the growth curves. (B,C) Bacterial lysates from A were exposed to SDS-PAGE (stained with Coomassie blue - B) and expression of BVR confirmed under the tested conditions by anti-HA Western blot (C). The predicted molecular weight of BVR is 35.82 kDa. Error bars represent ± one standard deviation, n = 3, and (*) denotes a significant (P≤0.05) difference between treated samples and solvent-treated samples. Infrared Fluorescent Protein (IFP) containing an HA tag (35.87 kDa) is shown in the rightmost lane as a positive control.
(TIF)

Figure S4 **Bilirubin increases the survival of EHEC in LPS-primed murine macrophages.** EHEC (86-24) cultured with solvent, biliverdin, bilirubin, or α-tocopherol, were exposed to either (A) J774A.1 murine macrophages or (B) LPS-primed J774.1 murine macrophages at an MOI of approximately 3. Bacteria remaining normalized to the amount of bacteria exposed to the macrophages. Error bars represent ± one standard deviation, n = 3, and (*) denotes a significant (P≤0.05) difference between treated samples and solvent-treated samples.
(TIF)

Figure S5 **Proteomic response of EHEC towards bile pigments.** EHEC (86-24) was cultured with solvent (NaOH, Sol), biliverdin (BV), or bilirubin (250 µM, BR) before proteomic analysis. In 2-D DIGE, the three conditions were separately labeled with different fluorophores, allowing for three comparisons of protein abundance from a single gel. For convenience, fluorescence is displayed as either green or red, and if equivalent amounts of fluoresces are apparent from each sample, the fluorescence is displayed as yellow. (A) Solvent-treated culture fluorescence is displayed as green while biliverdin-treated culture fluorescence is displayed as red. (B) Solvent-treated culture fluorescence is displayed as green while bilirubin-treated culture fluorescence is displayed as red. (C) Biliverdin-treated culture fluorescence is displayed as green while bilirubin-treated culture is displayed as red. (D) Five of the most prominently changed protein spots from each of the analytical methods. This experiment was conducted twice with similar results.
(TIF)

Figure S6 **Proteomically identified associated gene expression of EHEC when treated with bile pigments.** Isolated RNA from EHEC strain 86-24 was analyzed by qRT-PCR for transcriptional differences between genes associated with the proteins identified by our proteomic assay throughout the various treatment conditions. (A) Data of threshold cycle for housekeeping genes (normalized to total RNA) and (B) relative expression of associated genes (using *rpoS* as a reference gene) are displayed with solvent treated (white bars, normalized to 1.0 for standard expression), biliverdin treated (green bars) and bilirubin treated (yellow bars) conditions shown. The gene encoding the mannose specific transporter subunit (*manX*) was not included due to consistent issues with the PCR. Error bars represent ± one standard deviation, n = 3, and (*) denotes a significant (P≤0.05)difference between treated samples and solvent-treated samples.
(TIF)

Table S1 **Proteins identified in response to EHEC exposure to bilirubin.** Proteins identified through MALDI-TOF mass spectrometry. Percent volume ratio is averaged between two analyses.
(DOCX)

## References

[1] Everse J, Hsia N (1997) The toxicities of native and modified hemoglobins. Free Radic Biol Med 22: 1075–1099. 9034247 10.1016/s0891-5849(96)00499-6

[2] Vitek L, Ostrow JD (2009) Bilirubin chemistry and metabolism; harmful and protective aspects. Curr Pharm Des 15: 2869–2883. 19754364 10.2174/138161209789058237

[3] Maria Alexandra Brito RFMS, Dora Brites (2006) Bilirubin toxicity to human erythrocytes: A review. Clinica Chimica Acta 374: 46–56. 10.1016/j.cca.2006.06.012 16887110

[4] Ryter SW, Tyrrell RM (2000) The heme synthesis and degradation pathways: role in oxidant sensitivity. Heme oxygenase has both pro- and antioxidant properties. Free Radic Biol Med 28: 289–309. 11281297 10.1016/s0891-5849(99)00223-3

[5] Nishida T, Gatmaitan Z, Roy-Chowdhry J, Arias IM (1992) Two distinct mechanisms for bilirubin glucuronide transport by rat bile canalicular membrane vesicles. Demonstration of defective ATP-dependent transport in rats (TR-) with inherited conjugated hyperbilirubinemia. J Clin Invest 90: 2130–2135. 1430236 10.1172/JCI116098 PMC443282

[6] Leung JW, Liu YL, Leung PS, Chan RC, Inciardi JF, et al (2001) Expression of bacterial beta-glucuronidase in human bile: an *in vitro* study. Gastrointest Endosc 54: 346–350. 11522976 10.1067/mge.2001.117546

[7] Whiting JF, Narciso JP, Chapman V, Ransil BJ, Swank RT, et al (1993) Deconjugation of bilirubin-IX alpha glucuronides: a physiologic role of hepatic microsomal beta-glucuronidase. J Biol Chem 268: 23197–23201. 8226839

[8] Berk PD, Howe RB, Bloomer JR, Berlin NI (1969) Studies of bilirubin kinetics in normal adults. J Clin Invest 48: 2176–2190. 5824077 10.1172/JCI106184 PMC297471

[9] Hartmann F, Bissell DM (1982) Metabolism of heme and bilirubin in rat and human small intestinal mucosa. J Clin Invest 70: 23–29. 6806320 10.1172/JCI110598 PMC370221

[10] Stocker R (2004) Antioxidant activities of bile pigments. Antioxid Redox Signal 6: 841–849. 15345144 10.1089/ars.2004.6.841

[11] Dennery PA, McDonagh AF, Spitz DR, Rodgers PA, Stevenson DK (1995) Hyperbilirubinemia results in reduced oxidative injury in neonatal Gunn rats exposed to hyperoxia. Free Radic Biol Med 19: 395–404. 7590389 10.1016/0891-5849(95)00032-s

[12] Ching S, Ingram D, Hahnel R, Beilby J, Rossi E (2002) Serum levels of micronutrients, antioxidants and total antioxidant status predict risk of breast cancer in a case control study. J Nutr 132: 303–306. 11823595 10.1093/jn/132.2.303

[13] Grant DJ, Bell DA (2000) Bilirubin UDP-glucuronosyltransferase 1A1 gene polymorphisms: susceptibility to oxidative damage and cancer? Mol Carcinog 29: 198–204. 11170257

[14] Kapitulnik J (2004) Bilirubin: an endogenous product of heme degradation with both cytotoxic and cytoprotective properties. Mol Pharmacol 66: 773–779. 15269289 10.1124/mol.104.002832

[15] Rada B, Leto TL (2008) Oxidative innate immune defenses by Nox/Duox family NADPH oxidases. Contrib Microbiol 15: 164–187. 18511861 10.1159/000136357 PMC2776633

[16] Huycke MM, Abrams V, Moore DR (2002) *Enterococcus faecalis* produces extracellular superoxide and hydrogen peroxide that damages colonic epithelial cell DNA. Carcinogenesis 23: 529–536. 11895869 10.1093/carcin/23.3.529

[17] Jett BD, Huycke MM, Gilmore MS (1994) Virulence of enterococci. Clin Microbiol Rev 7: 462–478. 7834601 10.1128/cmr.7.4.462 PMC358337

[18] Schopfer P, Heyno E, Drepper F, Krieger-Liszkay A (2008) Naphthoquinone-dependent generation of superoxide radicals by quinone reductase isolated from the plasma membrane of soybean. Plant Physiol 147: 864–878. 18408044 10.1104/pp.108.118745 PMC2409040

[19] Nagaraja P, Avinash K, Shivakumar A, Dinesh R, Shrestha AK (2010) Simple and sensitive method for the quantification of total bilirubin in human serum using 3-methyl-2-benzothiazolinone hydrazone hydrochloride as a chromogenic probe. Spectrochim Acta A Mol Biomol Spectrosc 77: 782–786. 20829101 10.1016/j.saa.2010.08.003

[20] (2011) Outbreaks of *E. coli* O104:H4 infection: update 30. World Health Organization: Regional Office for Europe.

[21] Panzarini E, Inguscio V, Dini L (2011) Timing the multiple cell death pathways initiated by Rose Bengal acetate photodynamic therapy. Cell Death Dis 2: e169. 21654827 10.1038/cddis.2011.51 PMC3168993

[22] Serbinova E, Kagan V, Han D, Packer L (1991) Free radical recycling and intramembrane mobility in the antioxidant properties of alpha-tocopherol and alpha-tocotrienol. Free Radic Biol Med 10: 263–275. 1649783 10.1016/0891-5849(91)90033-y

[23] Guarner F, Malagelada JR (2003) Gut flora in health and disease. Lancet 361: 512–519. 12583961 10.1016/S0140-6736(03)12489-0

[24] Murray BE, Singh KV, Ross RP, Heath JD, Dunny GM, et al (1993) Generation of restriction map of *Enterococcus faecalis* OG1 and investigation of growth requirements and regions encoding biosynthetic function. J Bacteriol 175: 5216–5223. 8349561 10.1128/jb.175.16.5216-5223.1993 PMC204989

[25] Begley M, Gahan CG, Hill C (2005) The interaction between bacteria and bile. FEMS Microbiol Rev 29: 625–651. 16102595 10.1016/j.femsre.2004.09.003

[26] Arndt-Jovin DJ, Jovin TM (1989) Fluorescence labeling and microscopy of DNA. Methods Cell Biol 30: 417–448. 2467179 10.1016/s0091-679x(08)60989-9

[27] Silverman JA, Perlmutter NG, Shapiro HM (2003) Correlation of daptomycin bactericidal activity and membrane depolarization in *Staphylococcus aureus* . Antimicrob Agents Chemother 47: 2538–2544. 12878516 10.1128/AAC.47.8.2538-2544.2003 PMC166110

[28] O'Brien J, Wilson I, Orton T, Pognan F (2000) Investigation of the Alamar Blue (resazurin) fluorescent dye for the assessment of mammalian cell cytotoxicity. Eur J Biochem 267: 5421–5426. 10951200 10.1046/j.1432-1327.2000.01606.x

[29] Jansen T, Daiber A (2012) Direct Antioxidant Properties of Bilirubin and Biliverdin. Is there a Role for Biliverdin Reductase? Front Pharmacol 3: 30. 22438843 10.3389/fphar.2012.00030 PMC3306014

[30] Wilks A (2002) Heme oxygenase: evolution, structure, and mechanism. Antioxid Redox Signal 4: 603–614. 12230872 10.1089/15230860260220102

[31] McDonagh AF (2001) Turning green to gold. Nat Struct Biol 8: 198–200. 11224558 10.1038/84915

[32] Gourley GR (1997) Bilirubin metabolism and kernicterus. Adv Pediatr 44: 173–229. 9265971

[33] Dutt MK, Murphy GM, Thompson RP (2003) Unconjugated bilirubin in human bile: the nucleating factor in cholesterol cholelithiasis? J Clin Pathol 56: 596–598. 12890809 10.1136/jcp.56.8.596 PMC1770027

[34] Spivak W, DiVenuto D, Yuey W (1987) Non-enzymic hydrolysis of bilirubin mono- and diglucuronide to unconjugated bilirubin in model and native bile systems. Potential role in the formation of gallstones. Biochem J 242: 323–329. 3593251 10.1042/bj2420323 PMC1147708

[35] Vitek L, Jirsa M, Brodanova M, Kalab M, Marecek Z, et al (2002) Gilbert syndrome and ischemic heart disease: a protective effect of elevated bilirubin levels. Atherosclerosis 160: 449–456. 11849670 10.1016/s0021-9150(01)00601-3

[36] Cowger ML (1971) Mechanism of bilirubin toxicity on tissue culture cells: factors that affect toxicity, reversibility by albumin, and comparison with other respiratory poisons and surfactants. Biochem Med 5: 1–16. 5167173 10.1016/0006-2944(71)90069-x

[37] Shao C, Zhang Q, Sun Y, Liu Z, Zeng J, et al (2008) *Helicobacter pylori* protein response to human bile stress. J Med Microbiol 57: 151–158. 18201979 10.1099/jmm.0.47616-0

[38] Mustafa MG, Cowger ML, King TE (1969) Effects of bilirubin on mitochondrial reactions. J Biol Chem 244: 6403–6414. 4982202

[39] Flahaut S, Frere J, Boutibonnes P, Auffray Y (1996) Comparison of the bile salts and sodium dodecyl sulfate stress responses in *Enterococcus faecalis* . Appl Environ Microbiol 62: 2416–2420. 8779581 10.1128/aem.62.7.2416-2420.1996 PMC168024

[40] Crawford RW, Keestra AM, Winter SE, Xavier MN, Tsolis RM, et al (2012) Very long O-antigen chains enhance fitness during *Salmonella*-induced colitis by increasing bile resistance. PLoS Pathog 8: e1002918. 23028318 10.1371/journal.ppat.1002918 PMC3447750

[41] McDonagh AF, Lightner DA (1985) ‘Like a shrivelled blood orange’–bilirubin, jaundice, and phototherapy. Pediatrics 75: 443–455. 3883303

[42] Keshavan P, Schwemberger SJ, Smith DL, Babcock GF, Zucker SD (2004) Unconjugated bilirubin induces apoptosis in colon cancer cells by triggering mitochondrial depolarization. Int J Cancer 112: 433–445. 15382069 10.1002/ijc.20418

[43] Kotal P, Van der Veere CN, Sinaasappel M, Elferink RO, Vitek L, et al (1997) Intestinal excretion of unconjugated bilirubin in man and rats with inherited unconjugated hyperbilirubinemia. Pediatr Res 42: 195–200. 9262222 10.1203/00006450-199708000-00011

[44] Saxerholt H, Skar V, Midtvedt T (1990) HPLC separation and quantification of bilirubin and its glucuronide conjugates in faeces and intestinal contents of germ-free rats. Scand J Clin Lab Invest 50: 487–495. 2237261 10.1080/00365519009089163

[45] Vitek L, Majer F, Muchova L, Zelenka J, Jiraskova A, et al (2006) Identification of bilirubin reduction products formed by *Clostridium perfringens* isolated from human neonatal fecal flora. J Chromatogr B Analyt Technol Biomed Life Sci 833: 149–157. 10.1016/j.jchromb.2006.01.032 16504607

[46] Peters WH, Kock L, Nagengast FM, Kremers PG (1991) Biotransformation enzymes in human intestine: critical low levels in the colon? Gut 32: 408–412. 1902809 10.1136/gut.32.4.408 PMC1379081

[47] Torres AG, Payne SM (1997) Haem iron-transport system in enterohaemorrhagic *Escherichia coli* O157:H7. Mol Microbiol 23: 825–833. 9157252 10.1046/j.1365-2958.1997.2641628.x

[48] Mohawk KL, Melton-Celsa AR, Zangari T, Carroll EE, O'Brien AD (2010) Pathogenesis of *Escherichia coli* O157:H7 strain 86-24 following oral infection of BALB/c mice with an intact commensal flora. Microb Pathog 48: 131–142. 20096770 10.1016/j.micpath.2010.01.003 PMC2834854

[49] Cendrowski S, MacArthur W, Hanna P (2004) *Bacillus anthracis* requires siderophore biosynthesis for growth in macrophages and mouse virulence. Mol Microbiol 51: 407–417. 14756782 10.1046/j.1365-2958.2003.03861.x

[50] Campbell PA, Canono BP, Drevets DA (2001) Measurement of Bacterial Ingestion and Killing by Macrophages. Current Protocols in Immunology 14.6 doi:10.1002/0471142735.im1406s12 10.1002/0471142735.im1406s12 18432724

