# Supplementary figures and images for: A Product of Heme Catabolism Modulates Bacterial Function and Survival
Source: PLoS Pathog. 2013 Jul 25;9(7):e1003507. doi: 10.1371/journal.ppat.1003507 (PMC3723568; doi:10.1371/journal.ppat.1003507)

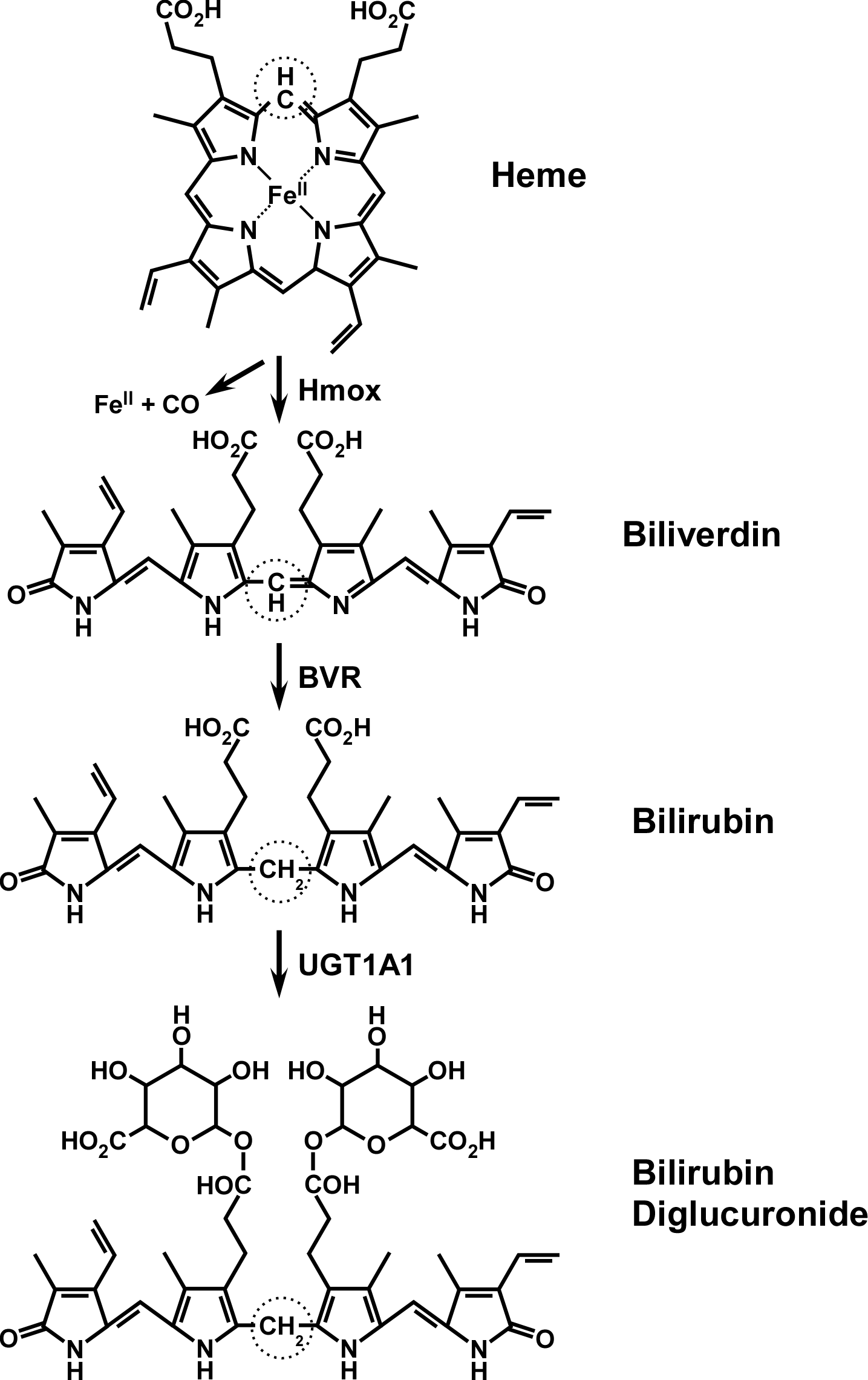

Supplement: Figure S1 — Chemical structure of heme catabolites. Heme is oxidatively cleaved by heme oxygenase (Hmox) to form ferrous iron (FeII), carbon monoxide (CO), and biliverdin. Biliverdin is further reduced by biliverdin reductase (BVR) to form the product bilirubin. Glucuronic acid molecules are conjugated to bilirubin by the UGT1A1 enzyme (expressed highly in the liver), forming bilirubin di-glucuronide, which can be easily passaged into bile [4]. Proposed reactive hydrogen(s) for neutralizing ROS though hydrogen donation are annotated within the dotted circles for each structure [10]. (TIF) [file ppat.1003507.s001.tif]

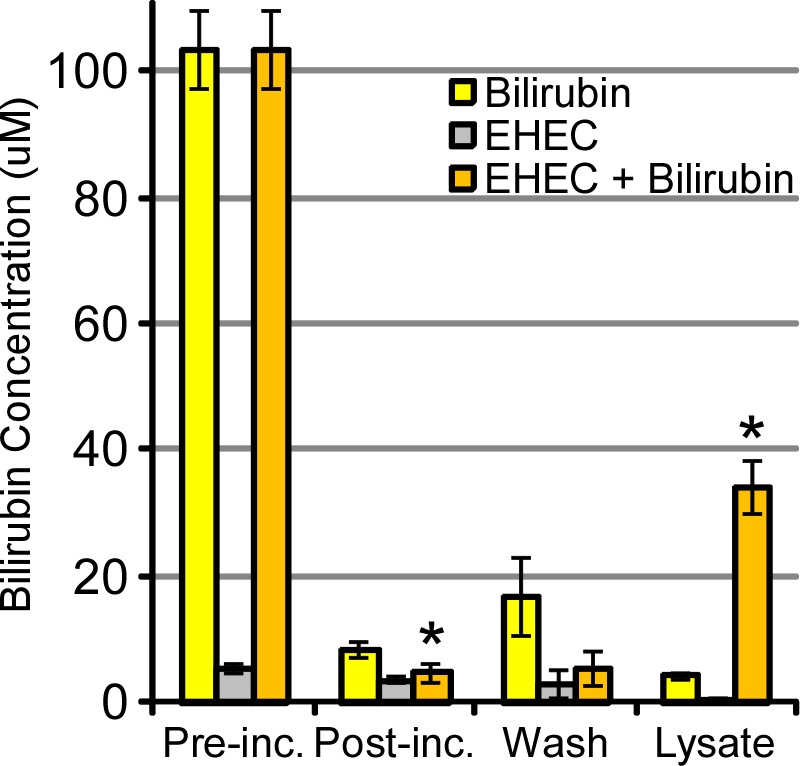

Supplement: Figure S2 — Association of bilirubin with EHEC upon exposure. EHEC strain 86-24 was incubated with or without bilirubin (approximately 100 µM) for 30 minutes, the cells washed twice with PBS prior to resuspention in ice cold milliQ water, and then sonicated for 12 seconds. Supernatants were collected prior to incubation (Pre-inc.), after incubation (Post-inc.), after the 1st wash (Wash), after the 2nd wash (not shown), and after lysis (Lysate). Bilirubin was quantified as described in Nagaraja et al. [19]. Error bars represent ± one standard deviation, n = 3, and (*) denotes a significant (P≤0.05) difference between EHEC and bilirubin samples and bilirubin alone samples. (TIF) [file ppat.1003507.s002.tif]

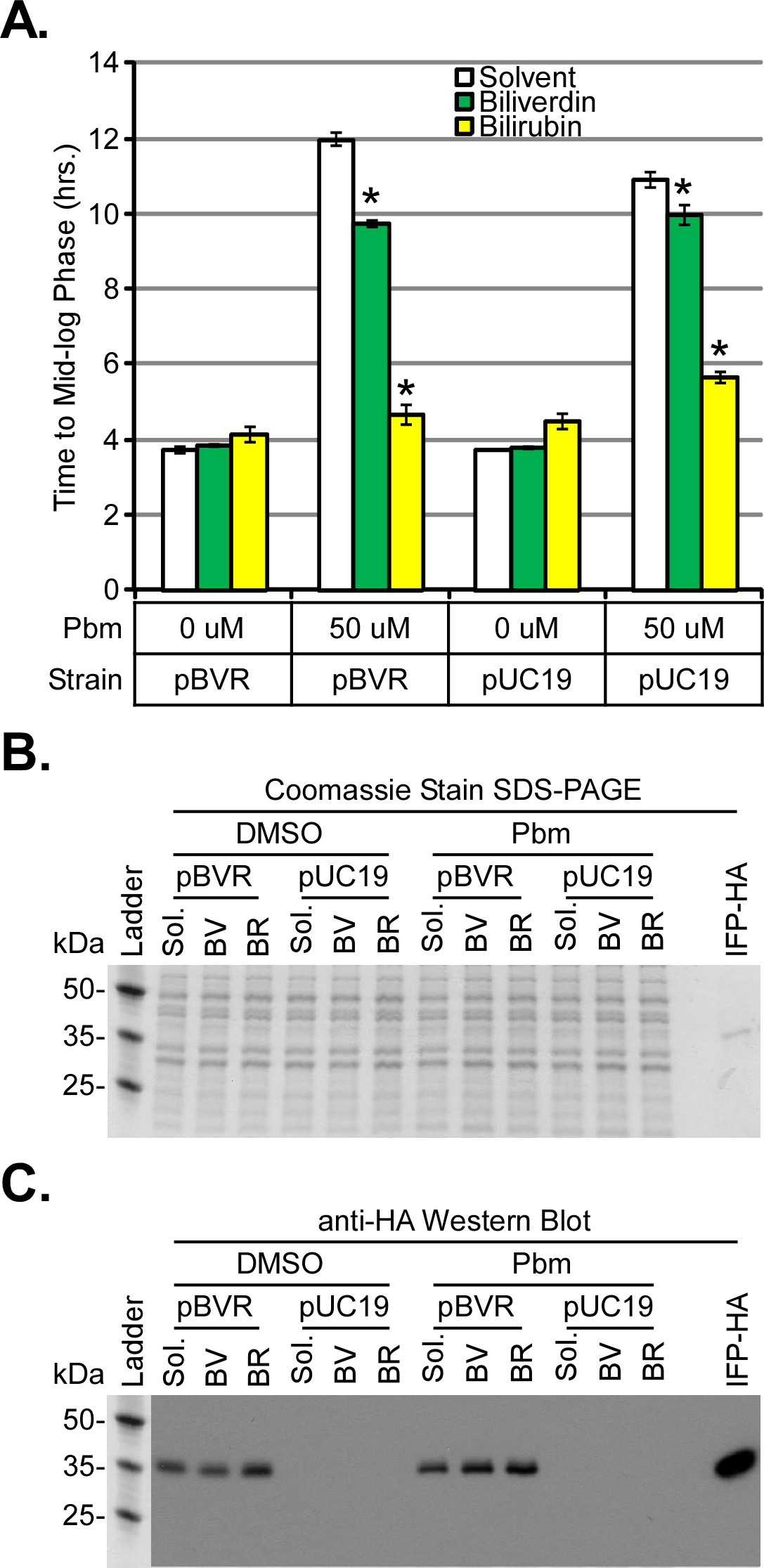

Supplement: Figure S3 — Expression of human BVR does not rescue EHEC from plumbagin-induced growth inhibition. (A) EHEC strain 86-24 containing the pUC19-BVR (pBVR) plasmid and EHEC strain 86-24 containing pUC19 were cultured with plumbagin (50 µM) to induce growth inhibition. Cultures were supplemented with biliverdin (500 µM, green bars) or bilirubin (500 µM, yellow bars) or solvent (NaOH, white bars). The time to mid-log phase was calculated from the growth curves. (B,C) Bacterial lysates from A were exposed to SDS-PAGE (stained with Coomassie blue - B) and expression of BVR confirmed under the tested conditions by anti-HA Western blot (C). The predicted molecular weight of BVR is 35.82 kDa. Error bars represent ± one standard deviation, n = 3, and (*) denotes a significant (P≤0.05) difference between treated samples and solvent-treated samples. Infrared Fluorescent Protein (IFP) containing an HA tag (35.87 kDa) is shown in the rightmost lane as a positive control. (TIF) [file ppat.1003507.s003.tif]

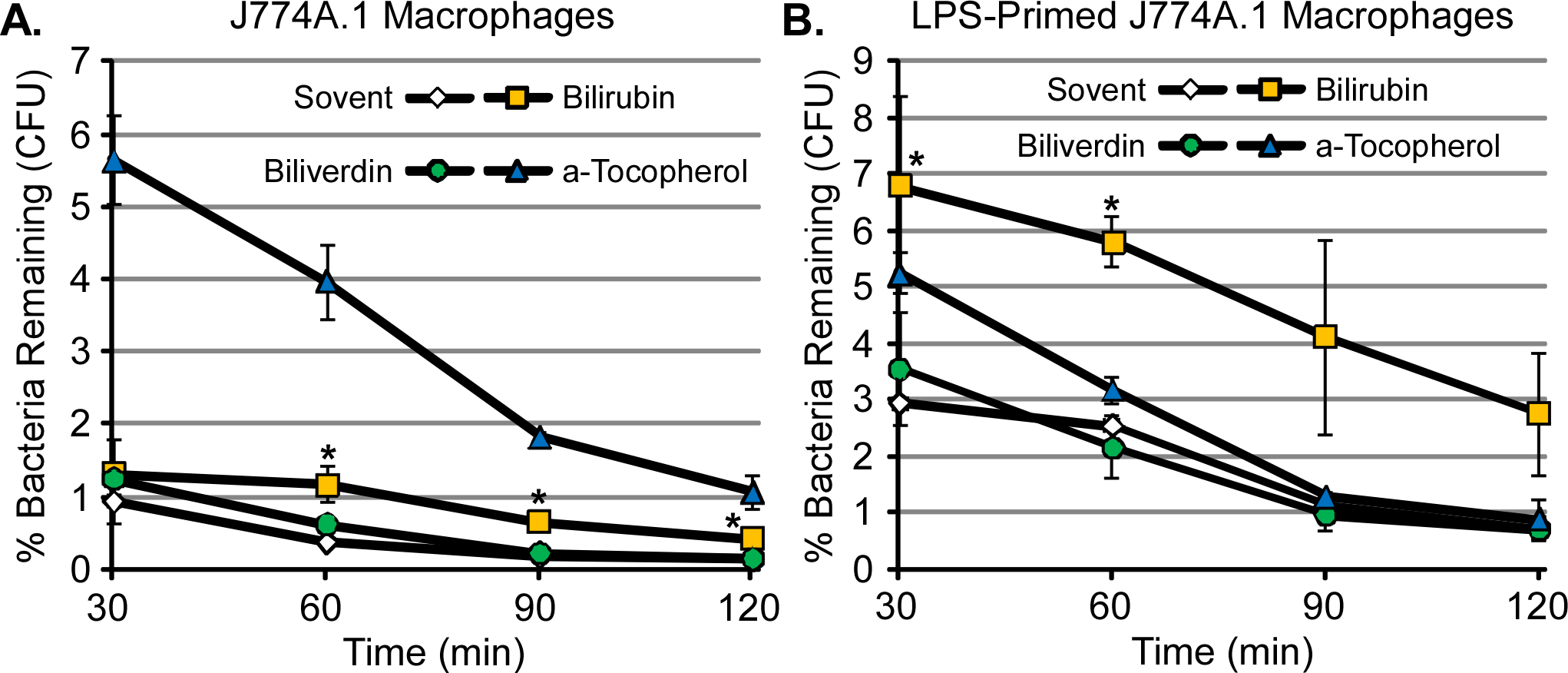

Supplement: Figure S4 — Bilirubin increases the survival of EHEC in LPS-primed murine macrophages. EHEC (86-24) cultured with solvent, biliverdin, bilirubin, or α-tocopherol, were exposed to either (A) J774A.1 murine macrophages or (B) LPS-primed J774.1 murine macrophages at an MOI of approximately 3. Bacteria remaining normalized to the amount of bacteria exposed to the macrophages. Error bars represent ± one standard deviation, n = 3, and (*) denotes a significant (P≤0.05) difference between treated samples and solvent-treated samples. (TIF) [file ppat.1003507.s004.tif]

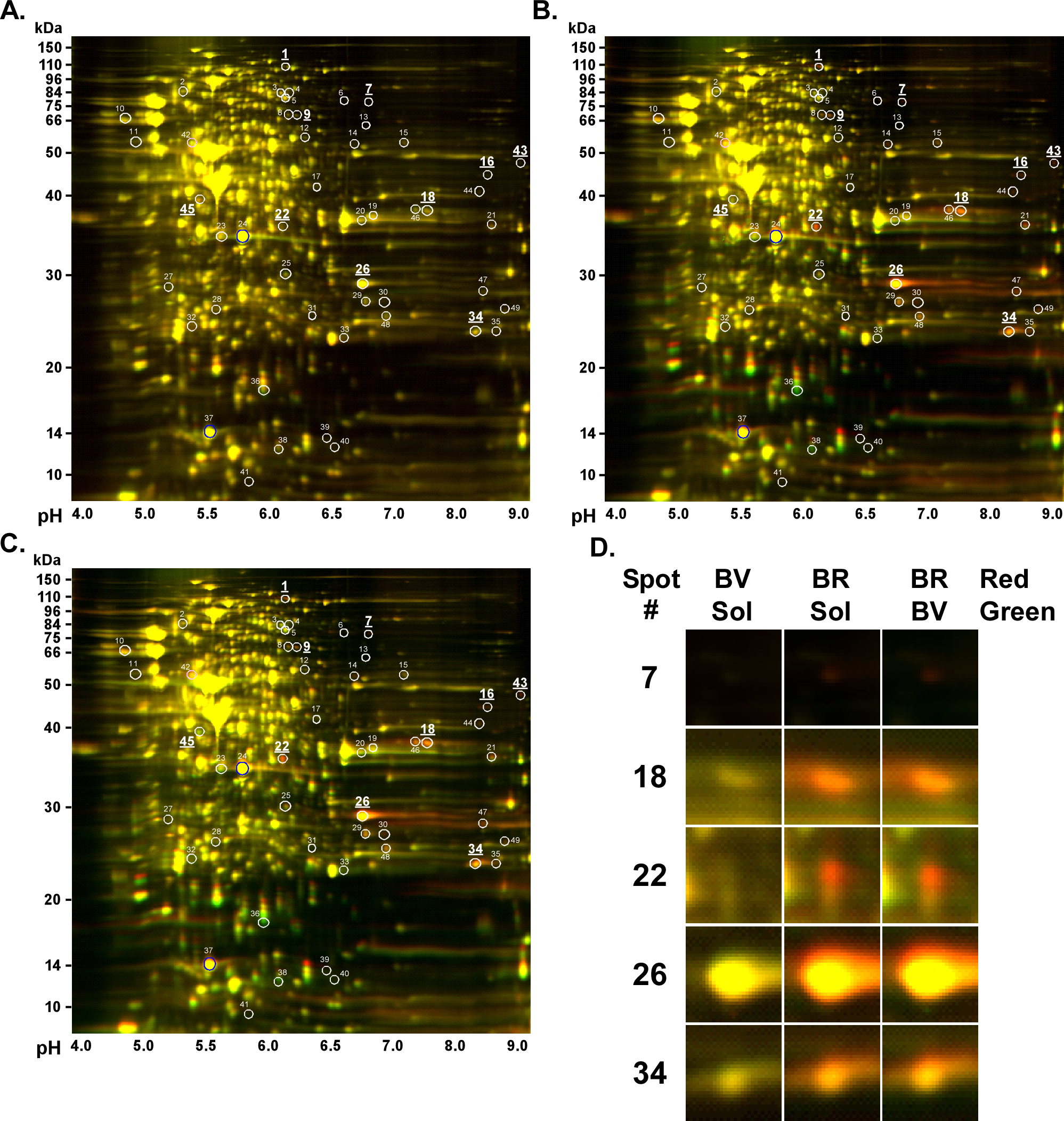

Supplement: Figure S5 — Proteomic response of EHEC towards bile pigments. EHEC (86-24) was cultured with solvent (NaOH, Sol), biliverdin (BV), or bilirubin (250 µM, BR) before proteomic analysis. In 2-D DIGE, the three conditions were separately labeled with different fluorophores, allowing for three comparisons of protein abundance from a single gel. For convenience, fluorescence is displayed as either green or red, and if equivalent amounts of fluoresces are apparent from each sample, the fluorescence is displayed as yellow. (A) Solvent-treated culture fluorescence is displayed as green while biliverdin-treated culture fluorescence is displayed as red. (B) Solvent-treated culture fluorescence is displayed as green while bilirubin-treated culture fluorescence is displayed as red. (C) Biliverdin-treated culture fluorescence is displayed as green while bilirubin-treated culture is displayed as red. (D) Five of the most prominently changed protein spots from each of the analytical methods. This experiment was conducted twice with similar results. (TIF) [file ppat.1003507.s005.tif]

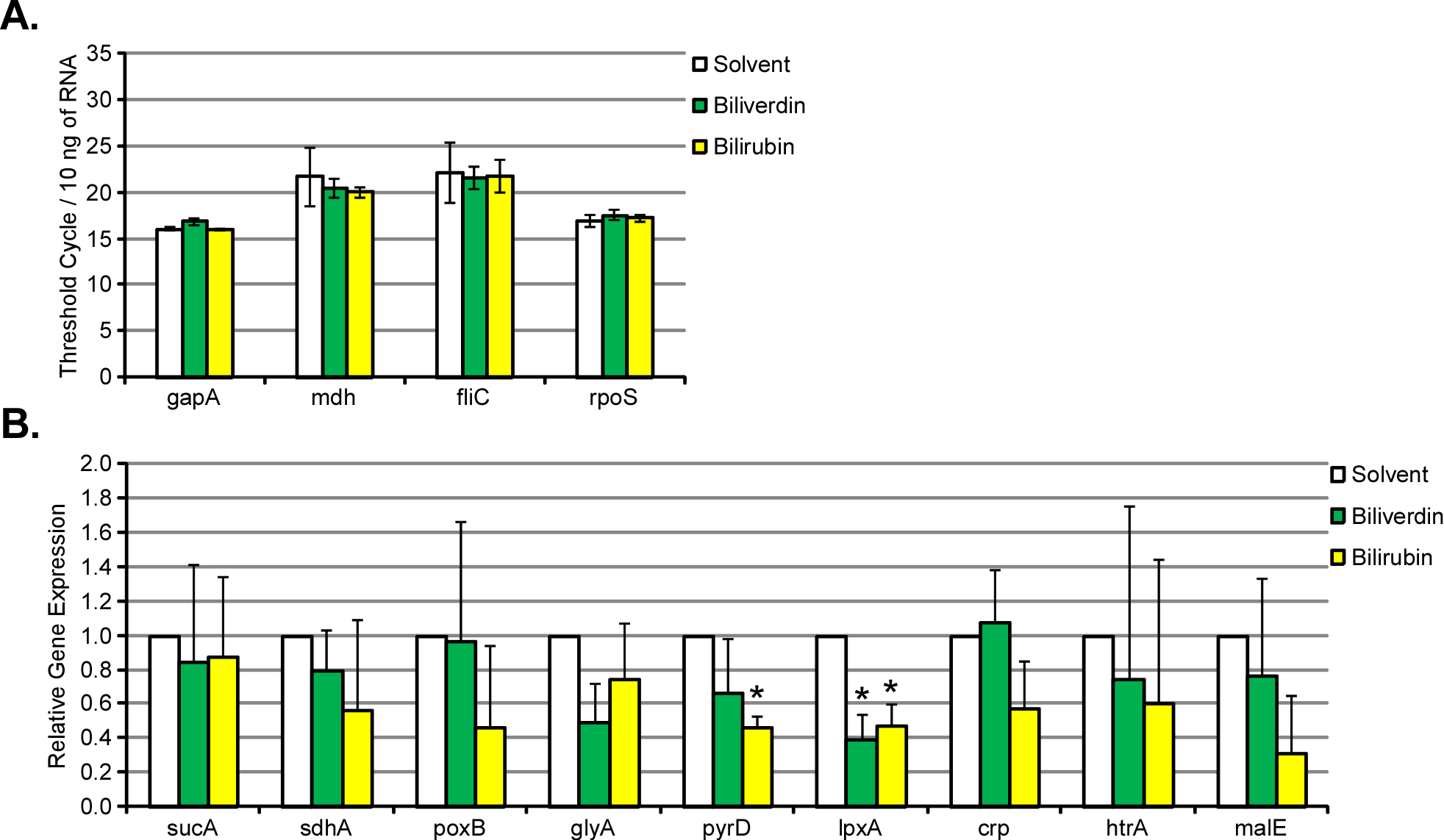

Supplement: Figure S6 — Proteomically identified associated gene expression of EHEC when treated with bile pigments. Isolated RNA from EHEC strain 86-24 was analyzed by qRT-PCR for transcriptional differences between genes associated with the proteins identified by our proteomic assay throughout the various treatment conditions. (A) Data of threshold cycle for housekeeping genes (normalized to total RNA) and (B) relative expression of associated genes (using rpoS as a reference gene) are displayed with solvent treated (white bars, normalized to 1.0 for standard expression), biliverdin treated (green bars) and bilirubin treated (yellow bars) conditions shown. The gene encoding the mannose specific transporter subunit (manX) was not included due to consistent issues with the PCR. Error bars represent ± one standard deviation, n = 3, and (*) denotes a significant (P≤0.05)difference between treated samples and solvent-treated samples. (TIF) [file ppat.1003507.s006.tif]
