# Supplementary material for: A Product of Heme Catabolism Modulates Bacterial Function and Survival
Source: PLoS Pathog. 2013 Jul 25;9(7):e1003507. doi: 10.1371/journal.ppat.1003507 (PMC3723568; doi:10.1371/journal.ppat.1003507)
Supplement: Table S1 — Proteins identified in response to EHEC exposure to bilirubin. Proteins identified through MALDI-TOF mass spectrometry. Percent volume ratio is averaged between two analyses. (DOCX) [file ppat.1003507.s007.docx]

**Supporting Table 1 – Proteins identified in response to EHEC exposure to bilirubin.**

| Functional Class | Spot No. | Protein (gene) | Assession No. | Associated  Gene | Peptide Count | Percentage Volume Ratio |
| --- | --- | --- | --- | --- | --- | --- |
| Metabolism | 1 | 2-oxoglutarate dehydrogenase E1 component | NP_286442 | *sucA* | 25 | 1.64 |
|  | 7 | succinate dehydrogenase flavoprotein subunit | NP_286439 | sdhA | 20 | 2.71 |
|  | 9 | pyruvate dehydrogenase | NP_286643 | poxB | 14 | 2.08 |
|  | 16 | serine hydroxymethyltransferase | NP_289107 | glyA | 20 | 2.41 |
|  | 22 | PTS system mannose-specific transporter subunit IIAB | NP_288253 | manX | 10 | 3.07 |
|  | 45 | maltose ABC transporter substrate-binding protein | NP_290668 | malE | 14 | -1.15 |
| Biosynthesis - Pyrimidine | 18 | dihydroorotate dehydrogenase 2 | NP_286820 | pyrD | 20 | 2.64 |
| Biosynthesis - LPS | 26 | UDP-N-acetylglucosamine acyltransferase | NP_285875 | lpxA | 5 | 2.86 |
| Gene Regulation | 34 | cAMP-regulatory protein | NP_289905 | crp | 14 | 2.39 |
| Proteolysis | 43 | serine endoprotease | NP_285857 | htrA | 18 | 2.06 |
